# Genomic Restructuring in the Tasmanian Devil Facial Tumour: Chromosome Painting and Gene Mapping Provide Clues to Evolution of a Transmissible Tumour

**DOI:** 10.1371/journal.pgen.1002483

**Published:** 2012-02-16

**Authors:** Janine E. Deakin, Hannah S. Bender, Anne-Maree Pearse, Willem Rens, Patricia C. M. O'Brien, Malcolm A. Ferguson-Smith, Yuanyuan Cheng, Katrina Morris, Robyn Taylor, Andrew Stuart, Katherine Belov, Chris T. Amemiya, Elizabeth P. Murchison, Anthony T. Papenfuss, Jennifer A. Marshall Graves

**Affiliations:** 1 Research School of Biology, The Australian National University, Canberra, Australia; 2 Department of Primary Industries, Parks, Water, and Environment, Prospect, Australia; 3 Department of Veterinary Medicine, University of Cambridge, Cambridge, United Kingdom; 4 Faculty of Veterinary Science, University of Sydney, Sydney, Australia; 5 Genome Resource Center, Benaroya Research Institute at Virginia Mason, Seattle, Washington, United States of America; 6 Cancer Genome Project, Wellcome Trust Sanger Institute, Cambridge, United Kingdom; 7 Bioinformatics Division, The Walter and Eliza Hall Institute for Medical Research, Parkville, Australia; 8 Department of Mathematics and Statistics, University of Melbourne, Melbourne, Australia; National Cancer Institute, United States of America

## Abstract

Devil facial tumour disease (DFTD) is a fatal, transmissible malignancy that threatens the world's largest marsupial carnivore, the Tasmanian devil, with extinction. First recognised in 1996, DFTD has had a catastrophic effect on wild devil numbers, and intense research efforts to understand and contain the disease have since demonstrated that the tumour is a clonal cell line transmitted by allograft. We used chromosome painting and gene mapping to deconstruct the DFTD karyotype and determine the chromosome and gene rearrangements involved in carcinogenesis. Chromosome painting on three different DFTD tumour strains determined the origins of marker chromosomes and provided a general overview of the rearrangement in DFTD karyotypes. Mapping of 105 BAC clones by fluorescence *in situ* hybridisation provided a finer level of resolution of genome rearrangements in DFTD strains. Our findings demonstrate that only limited regions of the genome, mainly chromosomes 1 and X, are rearranged in DFTD. Regions rearranged in DFTD are also highly rearranged between different marsupials. Differences between strains are limited, reflecting the unusually stable nature of DFTD. Finally, our detailed maps of both the devil and tumour karyotypes provide a physical framework for future genomic investigations into DFTD.

## Author Summary

The world's largest carnivorous marsupial, the Tasmanian devil, is threatened with extinction due to the emergence of devil facial tumour disease (DFTD), a fatal transmissible tumour. Critical loss of genetic diversity has rendered the devil vulnerable to transmission of tumour cells by grafting or transplanting the cells while biting and jaw wrestling. Initial studies of DFTD tumours revealed rearrangements among tumour chromosomes, with several missing chromosomes and four additional marker chromosomes of unknown origin. Since then, new strains of the disease have emerged and appear to be derived from the original strain. With no prior information available regarding the location of genes on normal devil chromosomes, a necessary first step towards characterisation of chromosome rearrangements in DFTD was to construct a map of the normal devil genome. This enabled us to elucidate the chromosome rearrangements in three DFTD strains. In doing so we determined the origin of the marker chromosomes and compared the three strains to determine which areas of the genome are involved in ongoing tumour evolution. Interestingly, rearrangements between strains are limited to particular genomic regions, demonstrating the unusual stability of this unique cancer. This study is therefore an important first step towards understanding the genetics of DFTD.

## Introduction

The Tasmanian devil (*Sarcophilus harrisii*), the world's largest extant carnivorous marsupial, was recently listed as an endangered species, primarily due to the emergence of a fatal, transmissible cancer known as devil facial tumour disease (DFTD) [1], [2]. Since the first reports of the disease in northeastern Tasmania in 1996, DFTD has rapidly spread to over 70% of the devil's range, causing population declines of around 90% in some regions [2], [3]. DFTD could lead to extinction of the species in the wild within 25–35 years [4]. Devils have a life expectancy of approximately six months from the first appearance of a lesion, with death occurring due to starvation, secondary infections and metastases [5]. In the absence of a vaccine or treatment for the disease, current measures taken to conserve the devil are focussed on breeding disease-free insurance populations in captivity [6]. Obviously, a deeper understanding of DFTD pathogenesis is required in order to help conserve this iconic species.

A striking feature of DFTD is that the tumour is a clonally derived cell line transmitted as an allograft between individuals by biting [7], [8], [9]. The only other example of a contagious cancer in the wild is Canine Transmissible Venereal Tumour (CTVT) in dogs, a histiocytic tumour typically transmitted through coitus [10]. Cytogenetic analysis of DFTD tumours from different individuals provided the first evidence of DFTD clonality. Pearse and Swift [8] demonstrated that DFTD tumours from 11 different individuals sampled from different locations in eastern Tasmania shared the same karyotype. This karyotype is highly rearranged, with loss of parts or all of three autosomes and the addition of four marker chromosomes. Since the description of the original DFTD karyotype, new karyotypic strains of the DFTD tumour have been identified, suggesting that the tumour is evolving [5]. G-banding shows that these strains are closely related, as would be expected of derivations of the original karyotype [5].

Determining the genome arrangement of normal devil chromosomes is an essential first step to characterising the rearrangements that have occurred in DFTD, identifying the genes that may have been altered by rearrangement and assessing how the tumour is evolving. However, prior to the emergence of DFTD very little cytogenetic analysis had been carried out on this species, and no molecular or mapping work.

The devil diploid karyotype consists of six pairs of autosomes and a pair of sex chromosomes (XX in females and XY in males). The devil belongs to the Family Dasyuridae, a group of marsupials renowned for their highly conserved 2n = 14 karyotypes [11], [12], [13]. Cross-species chromosome painting revealed homologous chromosome segments amongst even the most distantly related marsupials, including two dasyurid species, *Sminthopsis macroura* [14] and *Sminthopsis crassicaudata* [15]. These regions of homology can be applied to the devil karyotype enabling comparison with mapped and sequenced marsupial genomes, such as the South American opossum (*Monodelphis domestica*) [16] and the tammar wallaby (*Macropus eugenii*) [17], and allowing us to predict which genes will be present on each devil chromosome.

The origins of the marker chromosomes and the extent of rearrangement of tumour chromosomes are of intense interest. Initial G-band analysis of DFTD tumour chromosomes [8] showed that both copies of chromosome 1 and part of one copy of chromosome 2 (mislabelled as chromosomes 2 and 1 respectively in Pearse and Swift [8]), as well as both copies of chromosome 6 were replaced by marker chromosomes. However, this method is unable to detect molecular homology of marker chromosomes and lacks the resolution to determine the extent of rearrangement within the tumour karyotype. Chromosome painting of the tumour using whole chromosome probes generated from normal devil chromosomes, provides molecular information on the gross homologies between normal and DFTD chromosomes, although it cannot detect internal rearrangements. Gene mapping of normal and tumour cells provides information on changes in gene order, and detects rearrangements at a much higher resolution.

We have therefore employed these two complementary approaches to identify the origins of the DFTD tumour marker chromosomes and determine the extent of rearrangement between normal and DFTD chromosomes. We used chromosome painting to identify large regions of homology between normal and DFTD chromosomes. We mapped genes from the ends of opossum-wallaby evolutionary conserved gene blocks to identify chromosome homology on a finer scale. This allowed us to determine the origin of marker chromosomes and evaluate the differences between emerging strains of the disease. Our findings demonstrate the unusually stable nature of the tumour karyotype, even between strains, point to candidate genes involved in tumourigenesis and indicate that certain regions of the genome are hotspots for rearrangement in marsupial evolution and in DFTD tumours. This information provides a framework for studies of genome changes at the sequence level that underlie the transmissible tumour in the Tasmanian devil.

## Results

We constructed a gene map of the normal devil genome by mapping 105 bacterial artificial chromosome (BAC) clones containing genes from the ends of opossum-wallaby conserved gene blocks, providing information on the arrangement of the normal devil genome. We then used a combination of chromosome painting and gene mapping to determine the rearrangements which have occurred between the normal and DFTD tumour genomes. Chromosome paints from the normal genome painted onto DFTD chromosomes from three different tumour strains provide insight into the gross rearrangements which have occurred to result in the DFTD tumour and indicate the origin of marker chromosomes. Gene mapping of 105 genes on DFTD chromosomes from these three strains further refined the extent of rearrangement in DFTD tumour strains.

### Physical map of normal devil genome

To efficiently construct the physical map, conserved blocks of genes were identified by comparing the anchored opossum genome assembly to the physical map of the tammar wallaby genome. A total of 60 opossum/wallaby conserved gene blocks were identified, covering the entire genome. Genes located near the ends of these conserved gene blocks were used to search the devil transcriptome [7] and overgos were designed using devil sequence to isolate BACs for these genes from a male 6.5× genome coverage devil BAC library (VMRC-50) with an average insert size of approximately 140 kb. These BACs were mapped onto normal devil male metaphase chromosomes. Smaller conserved gene blocks (estimated from the opossum genome to be less than 4 Mb) were localized by mapping a single gene, rather than genes from either end of the block, due to the limitations of FISH to accurately determine the orientation of BACs that are so close together.

**Figure 1:**
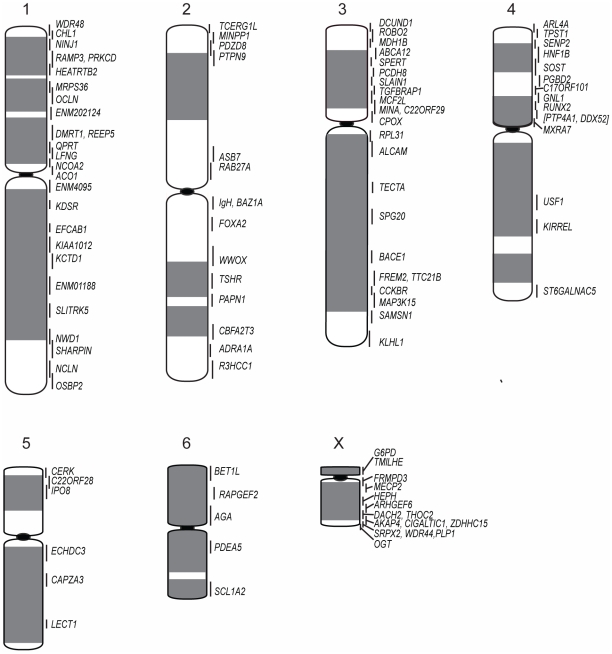
Physical map and ideogram of the devil genome. The DAPI banding pattern for each chromosome is shown in grey. Genes contained within the same BAC clone are indicated in brackets. Chromosomes have been arranged in order according to two previously published karyotypes [19], [20], which differs from the karyotype presented by Pearse and Swift [8] in the order of chromosomes 1 and 2.

The resulting physical map contains 105 genes (see Table S1 for a list of genes and their corresponding BACs). Chromosomes 1, 3 and X are the most densely mapped. Few genes have been mapped to chromosome 5 (Figure 1; Table 1), even after new and/or redesigned overgos were used to screen the BAC library (see Table S2 for details on overgo success rate). The low success rate for chromosome 5 suggests that clones from this chromosome may be under-represented in the library, perhaps due to a lack of EcoRI fragments of the size selected for library construction.

**Table 1:** Number of genes mapped to each normal devil chromosome.

Chromosome	Number of Genes	Chromosome Length(% of haploid female genome)
1	26	22
2	15	21
3	23	20
4	15	16
5	6	10
6	5	8
X	15	3
Total	105	

Cross-species chromosome painting was used to predict which devil chromosome would contain each of the genes. Although devil chromosomes have not been used for such cross-species hybridisations, chromosome painting has been performed on two other dasyurid species (*Sminthopsis crassicaudata* and *S. macroura*) [14], [15]. As the dasyurid karyotype is highly conserved, we could use data from these two species to make predictions in the devil. Of the 105 genes mapped, only four *(ARL4A, BET1L, LECT1, SLITRK5*) mapped to unexpected locations. Sequencing was used to confirm that these four BACs did contain the gene of interest (Figure S1). Overall, the gene mapping data correlated with cross-species chromosome painting on *Sminthopsis* species and extends the observation of a highly conserved dasyurid karyotype.

One discrepancy between the reported painted [14], [15], [18] and G-banded karyotypes [19] and the karyotype described by Pearse and Swift [8] is whether chromosome 1 is the large metacentric or submetacentric chromosome. Here we used the long-established classification of Martin and Hayman [11], [20], which was subsequently used in classic comparisons with other marsupial karyotypes [12] and in chromosome painting studies [14], [15], designating chromosome 1 as the large submetacentric chromosome in dasyurids, corresponding to conserved segments C1 to C6 based on chromosome painting [15]. Chromosome 2 consists of conserved segments C7, C8 and C9 [15].

**Figure 2:**
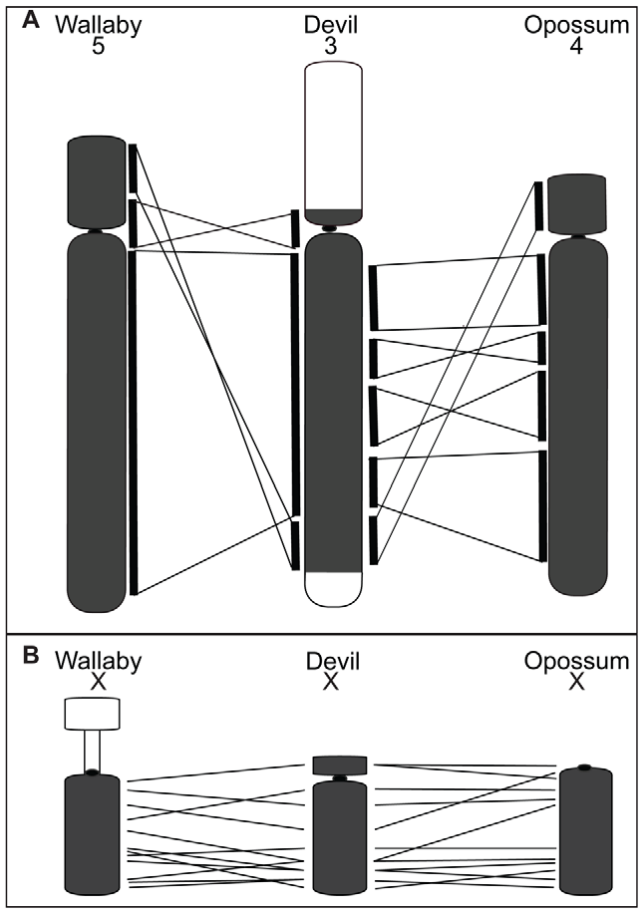
Comparison of gene arrangement among devil, wallaby, and opossum chromosomes. (A) Gene order for the region shaded in grey on devil chromosome 3 is well conserved between wallaby chromosome 5 and opossum chromosome 4. The white regions on devil chromosome 3 are homologous to wallaby chromosome 6 and opossum chromosome 7 (Figure S2). (B) A comparison of gene order on the devil X chromosome with wallaby and opossum X chromosomes, where extensive reshuffling of gene order is evident.

Although chromosome painting confirms that even distantly related marsupials share large regions with DNA homology [14], [15], [18], our comparison between gene arrangement in the devil, opossum and wallaby shows that within some of these blocks, gene order has been highly rearranged by multiple inversions (Figure 2). The most conserved chromosome amongst the marsupials was the long arm of devil chromosome 3 (Figure 2A, the short arm of devil chromosome 3 corresponds to wallaby chromosome 6 and opossum chromosome 7, Figure S2A), which appears as a single block conserved between the wallaby and devil, although there have been two inversions in this region with respect to the opossum. Highly rearranged chromosomes include the devil X chromosome (Figure 2B) and chromosome 1. Chromosomes 2 and 4 show an intermediate level of rearrangement (Figure S2B and S2C). Too few genes were mapped to chromosomes 5 and 6 to determine the extent of conservation or rearrangement between species. By mapping the ends of opossum/wallaby conserved gene blocks we hoped that we could virtually assign each gene within these conserved gene blocks to a location on devil chromosomes. The extent of rearrangement between these three species makes the construction of a virtual map based on both gene content and gene order difficult, and would require the localization of many more genes. However, we are able to predict the gene content of each block and hence, the gene content of each chromosome.

### Chromosome painting on DFTD tumour cell line strains

Since the DFTD karyotype was first reported in 2006, multiple karyotypic ‘strains’ have been discovered [5]. The various strains are characterized by minor cytogenetic rearrangements that demonstrate ongoing tumour evolution as the disease spreads across Tasmania. Only a small number of readily identifiable rearrangements distinguish the three strains; the basic composition of the DFTD karyotype is preserved. The random gains, losses and translocations that characterize unstable tumour karyotypes are not present in any of the DFTD strains, which are therefore considered stable. The three strains are readily identifiable with G-banding; however, this technique is insufficiently precise to determine the genomic regions that are specifically rearranged in each strain. The comparatively finer technique of chromosome painting permitted a more detailed characterisation of the DFTD karyotype, as well as the progressive chromosome changes that distinguish three tumour strains.

Chromosome painting using whole chromosome probes derived from normal devil chromosomes (see Figure S3 for the flow karyotype) was carried out on eight tumour samples comprising Strains 1, 2 and 3. Samples were collected from animals at different locations throughout Tasmania (refer to Figure S4 for strain details). The diagnostic DFTD karyotype is present in all tumours, with only subtle cytogenetic differences between strains. All DFTD cell lines were karyotypically stable in cell culture, with no progressive chromosome rearrangements detected after multiple (greater than 10) passages. Thus the tumour karyotype was found to be remarkably stable *in vivo* and *in vitro*, with only minor cytogenetic differences between strains, a surprising result considering the rapid proliferation and malignant behaviour of neoplastic cells.

**Figure 3:**
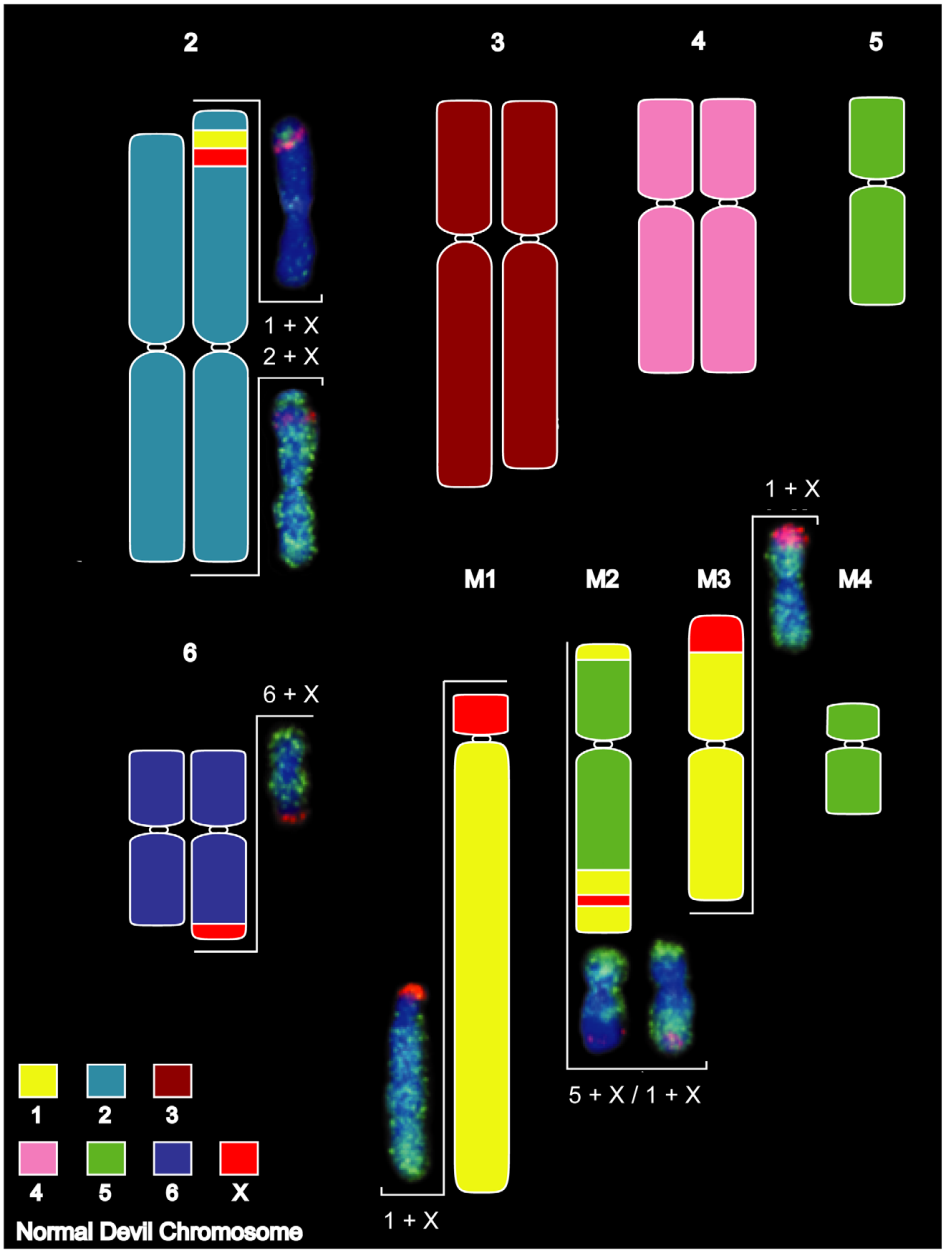
Summary of chromosome painting results for DFTD Strain 1. DFTD chromosomes have been colour-coded to reflect their homology to normal devil chromosomes. Two-colour FISH painting results are shown for the X chromosome (red) and autosomes 1, 2, 5 and 6 (green).

Painting of cells from DFTD Strain 1 revealed that the four marker chromosomes were derived predominantly from chromosomes 1, 5 and X (Figure 3). The giant marker chromosome (M1) consisted almost entirely of chromosome 1 material, which also made smaller contributions to markers 2 and 3 (M2 and M3, respectively), as well as a small insertion in chromosome 2p. The chromosome 5 probe hybridised to the single copy of chromosome 5 present in the tumour, as well as to M2 and M4 in relatively simple rearrangements.

X chromosome rearrangements were more complex, with small insertions of X material in M2 and chromosome 2p, adjacent to the chromosome 1 insertion, and extensive rearrangement between chromosomes 6q, M1 and M3. The Y chromosome could not be detected within the tumour using a probe generated by manual microdissection (Figure S5), suggesting that the original tumour derived from a normal cell of a 2X female.

**Figure 4:**
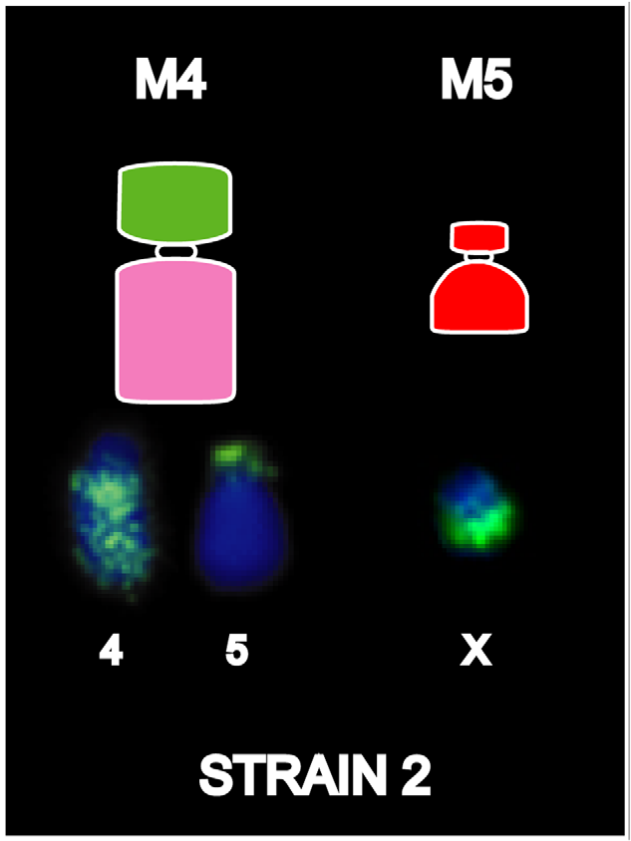
Chromosome painting results unique to DFTD strain 2. Differences detected between Strains 1 and 2 are limited to the detection of chromosome 4 on M4 and X chromosome on an additional marker chromosome, M5.

Based on both G-banding and chromosome painting results, strain 1 cells were found to retain the basic DFTD karyotypic framework, whereas Strains 2 and 3 were marked by additional rearrangements. In Strain 2 and a proportion of Strain 3 tumours, an additional marker chromosome M4 was hybridized by the chromosome 4 paint throughout the long arm, and an additional reciprocal translocation between chromosomes 4 and 5 (Figures S6 and S7). These strains had an additional marker chromosome M5, which completely hybridised to the X paint (Figure 4, Figure S6).

Strain 3 karyotypes were found to be somewhat more complicated than for Strains 1 and 2, showing variation of painting patterns between tumour cell lines isolated from different animals, and the presence of two distinct sub-strains in two of the three tumours examined. M4 was variably present in Strain 3 tumours, with loss of this marker in 0–64% of metaphases in different tumour cell lines (see Figure S6). The variable loss of M4 was interpreted as a relatively minor change and was not considered indicative of more broad scale karyotypic instability. Strain 3 karyotypes were otherwise similar to those of Strain 2, with the exception of chromosomes 4 and 5, which were further rearranged in a proportion of tumours. Figure S6 catalogues the chromosome 4 and 5 rearrangements unique to Strain 3 tumours and compares the sub-strains present in two of the tumours examined. An additional translocation between chromosomes 4p and M4q was present in some cells in Strains 3A and 3C. This translocation was present in all metaphases of Strain 3A, compared with only 12.5% (1 out of 8) of Strain 3C metaphases, and was absent in Strain 3B. Strain 3B also exhibited some heterogeneity; 36% of cells lacked M5 (18 out of 50), and 58% (29 out of 50) lacked M4. In cells lacking M4, the chromosome 5 paint hybridised to the short arm of the giant marker, replacing the X chromosome signal present at this location in all other tumours. In the 36% (18/50) of tumours that had M4, the chromosome 5 paint hybridised to the long arm of M4, as for Strain 1 tumours.

Paints generated from flow-sorted normal devil chromosomes have therefore revealed the origin of the genomic material that comprises each marker chromosome, as well as several insertions undetectable with G-banding. Painting also demonstrated the extent to which chromosomes 1, 4, 5 and the X chromosome are rearranged in DFTD. None of this information could be gained from earlier G-banding studies. Our findings indicate that progressive rearrangements of chromosomes 4, 5 and the X chromosome distinguish the three strains, and that multiple Strain 3 tumours are composed of at least two sub-strains, present in varying proportions, implying that passage of the tumour from animal to animal is usually via multiple cells.

### Physical map of DFTD tumour cell strains

**Figure 5:**
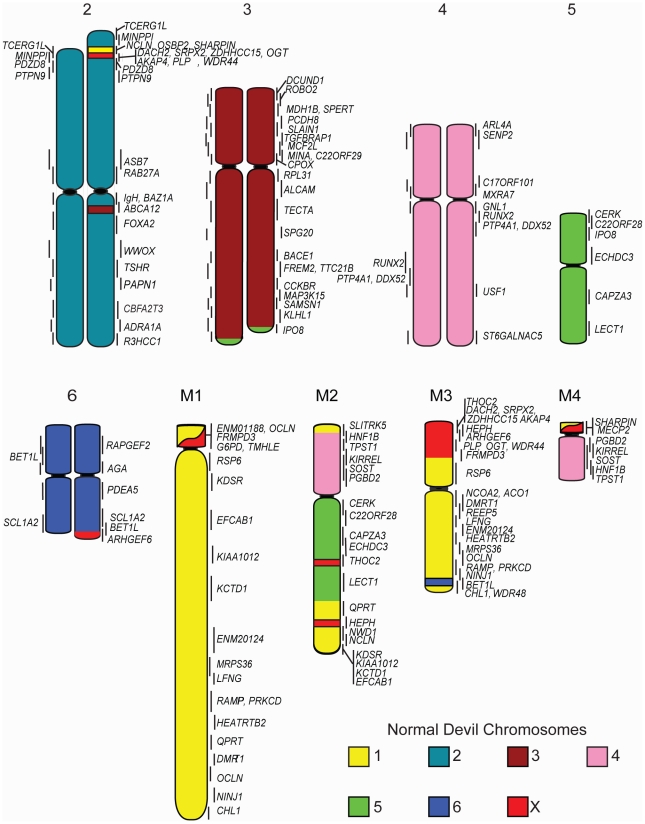
Physical map of DFTD Strain 1. Chromosomes have been coloured coded to reflect their homology to normal devil chromosomes. For chromosomes 2, 3, 4, and 6, gene names are indicated for each homologue to highlight differences in gene order or position between homologues. Despite a size difference between the two homologues of chromosome 3, gene order is the identical.

The resolution afforded by painting is insufficient to identify the genetic constitution of breakpoints associated with tumour cell rearrangements. To pinpoint rearrangements in the DFTD tumour, we therefore constructed a physical map of the three tumour strains described above, using the same 105 genes we used to construct the physical map of the normal devil genome. This map of the tumour genomes (Figure 5) shows that rearrangement in the tumour has been more extensive than could be detected by chromosome painting (Figure S8).

**Figure 6:**
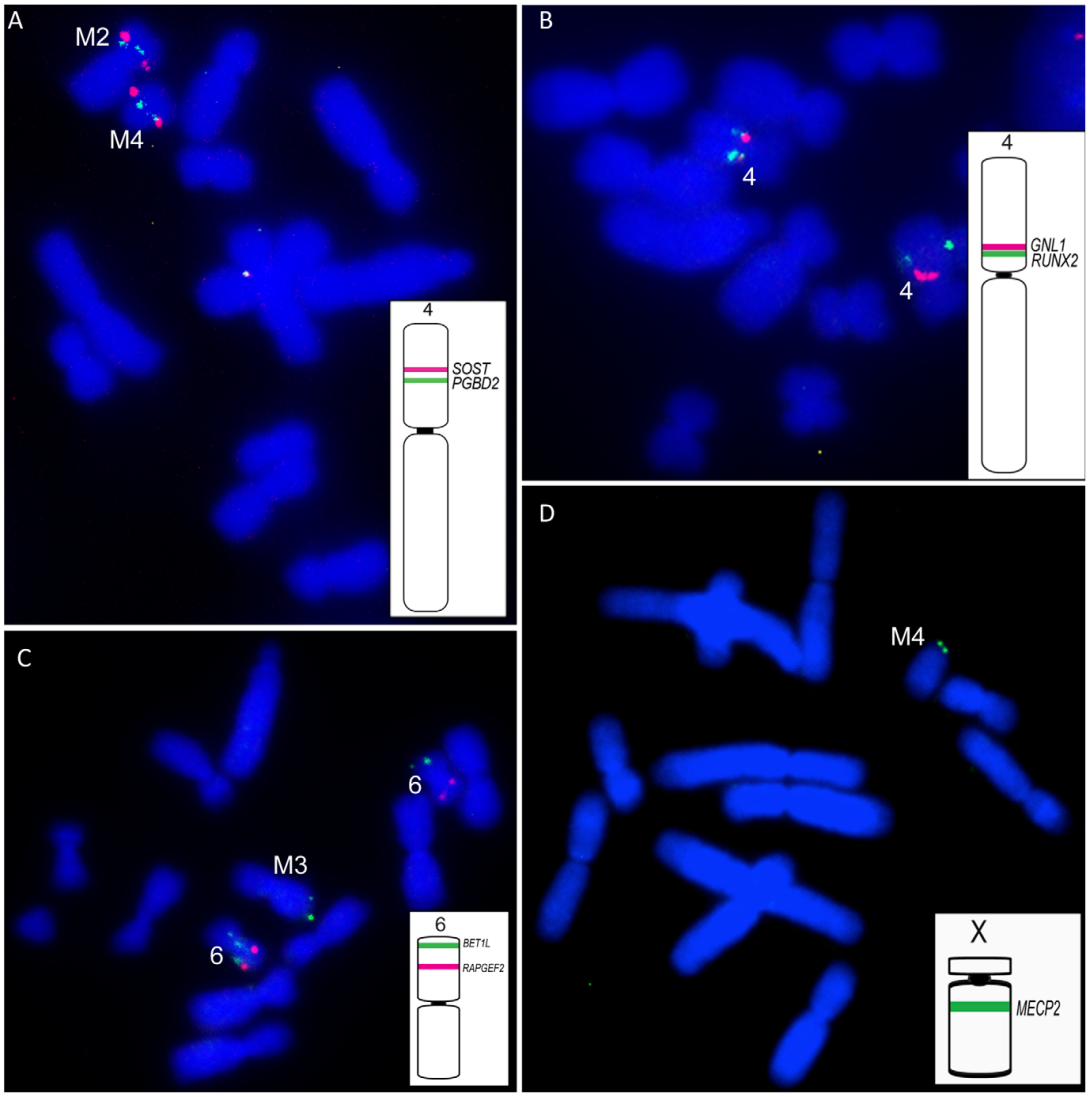
Representative FISH results on DFTD chromosomes. In each case an ideogram of the location of genes on the normal devil chromosome are indicated, with the colour of the line on the normal chromosome corresponding to the colour of the FISH signals on DFTD chromosomes (A) *SOST* and *PGBD2* located on normal devil chromosome 4 localise to M2 and M4 in DFTD. (B) *GNL1* and *RUNX2* located on the short arm of normal devil chromosome 4, map to the long arm of DFTD chromosome 4, with *GNL1* proximal and *RUNX2* mapping to different locations on each homologue. (C) Chromosome 6 genes *BET1L* and *RAPGEF2*. *BET1L* has an additional copy in DFTD, mapping to different locations on the two copies of chromosome 6 as well as distal M3. (D) The X-borne gene *MECP2* is detected in DFTD, mapping to the short arm of M4.

Genes from chromosome 1 were found on one copy of distal 2p, the long arm of M1, distally on both arms of M2 and much of M3, as was also indicated by chromosome painting. In addition, gene mapping demonstrated the presence of chromosome 1 genes on the short arm of M1 and M4. Gene mapping also revealed an addition of at least one chromosome 3 gene (*ABCA12*) to the long arm of one copy of chromosome 2, and the addition of at least one chromosome 5 gene (*IPO8*) to the long arm of chromosome 3 (Figure 5). Four of the 12 genes mapped to the short arm of chromosome 4 are found on the short arm of M2 and long arm of M4 (e.g. *SOST* and *PGBD2*, Figure 6A). Repositioning of the centromere was also detected, and reordering of many of the genes remaining on chromosome 4 in the tumour (e.g.*GNL1* and *RUNX2*) (Figure 6B). Gene *BET1L* mapped to different locations on the two homologues of chromosome 6 and another copy of *BET1L* was found to be located on M3 (Figure 6C).

As predicted by chromosome painting, X chromosome genes were located on one homologue of 2p, one homologue of chromosome 6, the short arm of M1, distal M2q and proximal M3. In addition, at least one X-borne gene (*MECP2*) was found on the short arm of M4 (Figure 6D).

Both painting and mapping data identified two copies of chromosome 6 present in DFTD and one intact copy of chromosome 5 with the other copy distributed across marker chromosomes, conflicting with the original DFTD karyotype reported by Pearse and Swift [8]. Given the reshuffling of gene order and the addition of a region from the X chromosome inserted on one homologue of chromosome 6, it is not surprising that the identity of this chromosome could not be accurately determined by G-banding. Likewise, the size difference between the two large metacentric chromosomes was initially interpreted as a deletion of part of the long arm on one homologue. However, our gene mapping shows that the size differences between the two copies of chromosome 2 are due to addition to the short arm of one homologue of regions bearing genes from chromosomes X and 1.

**Figure 7:**
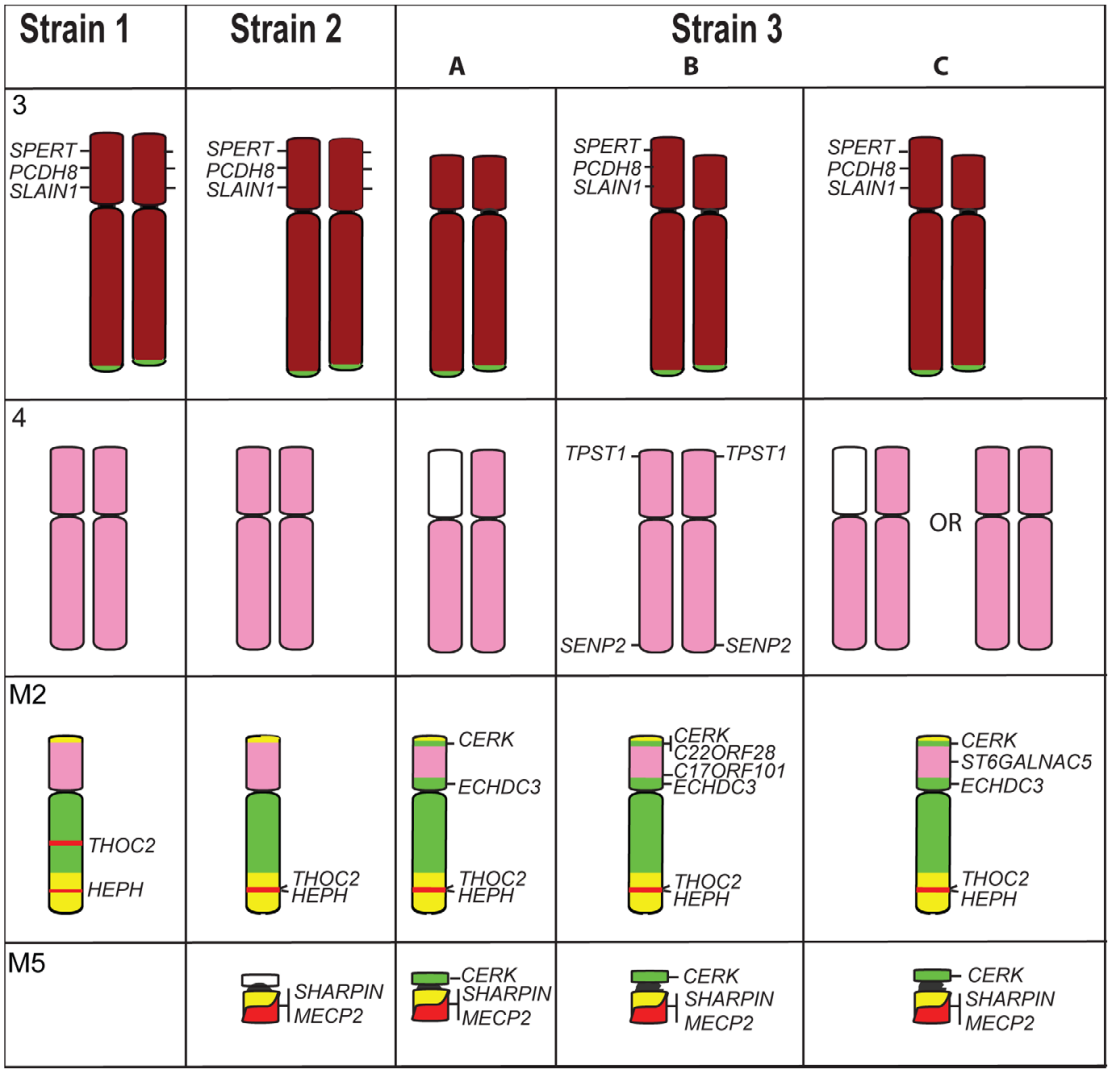
Differences detected by gene mapping among Strains 1, 2, and the three different Strain 3s. Genes *SPERT*, *PCDH8* and *SLAIN1* are found on both homologues of chromosome 3 in Strains 1 and 2 (gene names are only indicated next to one homologue) but a deletion of these genes has occurred on both homologues of Strain 3A, and one homologue of Strain 3B and 3C. Chromosome 4, which appears identical between Strains 1 and 2, is different among the Strain 3s. In Strain 3A, genes mapped only to the short arm of one copy of chromosome 4. Strain 3B has retained *TPST1* on 4p, a gene mapping to M2 and M4 in all other strains, and the 4p *SENP2* gene, has translocated to 4q. X chromosome genes *THOC2* and *HEPH* map to different location on M2 in Strain 1 but colocalise in other strains. Strains 2 and 3 have an additional marker chromosome (M5), which contains *SHARPIN* and *MECP2* in Strain 2 and 3, as well as *CERK* in Strain 3. Colour coding of chromosomes is the same as that used in Figure 3.

Confirming our results from chromosome painting, gene mapping revealed only subtle differences between tumour strains. The additional marker chromosome (M5) of Strain 2 was found to contain one gene from the X chromosome (*MECP2*) and one gene from chromosome 1 (*SHARPIN*). The only other detectable difference between Strains 1 and 2 is the location of X chromosome genes *HEPH* and *THO2C*, which were observed to be near to each other, but not adjacent, in the normal devil genome (Figure 7). In Strain 2 they mapped to the same location on M2, but in Strain 1, they were found to be separated by chromosome 1 and 5 genes.

A readily distinguishable difference between G-banded karyotypes of tumour strains was found to be the deletion of part of the short arm of chromosome 3 uniquely in Strain 3. We have confirmed this by gene mapping and show that the region deleted spans from *MDH1B* on distal 3p to *TGFBRAP1* on proximal 3p. Only one copy of the chromosome has this deletion in strain 3B, but both copies have the deletion in Strain 3A (Figure 7) and no signals were observed for these genes on any other chromosome, suggesting these genes are completely absent from the tumour genome. The deletions appeared to be the same on both copies of chromosome 3, suggesting that the normal member of the pair may have been lost, and the deleted copy reduplicated.

The three Strain 3 tumours also have variations in the arrangement of chromosome 4 and 5 genes (Figure 7). Genes from the short arm of chromosome 4 were observed to be absent from one copy of the chromosome in Strain 3A, and this deletion is also present in 20% of Strain 3C metaphase spreads. In addition, Strain 3B was found to have retained *TPST1*and *SENP2* on chromosome 4 (these genes were found on M4 in all other strains), although *SENP2* was observed to be translocated to 4q. This strain was shown also to have acquired an additional copy of *C17orf101* on the short arm of M2. Strain 3C had three copies of *ST6GALNAC5*, one copy on each of the chromosome 4 homologues observed in all strains, as well as an additional copy on the short arm of M2. In all three Strain 3s, chromosome 5 genes were detected on the short arm of M2, and in strain 3C also on M5.

Gene mapping can also detect variation in the numbers of copies of a gene, revealing a copy number increase or deletion of small regions of the genome that are hard to detect by chromosome painting. Nearly all genes mapped in the tumour were observed to be present in two copies, but for Strain 1, we identified twelve autosomal genes present in only one copy and three autosomal genes present in three copies (Table 2). Significantly, we found that 11 of the 14 genes from the X chromosome were present in two copies, consistent with the origin of the original tumour from an XX female.

**Table 2:** Genes deleted or increased in copy number in DFTD.

Gene	Normal	DFTD Tumour (Strain 1)
**Deleted**		
*ACO1*	1p	M3q
*NCOA2*	1p	M3q
*REEP5*	1p	M3
*WDR48*	1p	M3
*ENM01188*	1q	M1
*NWD1*	1q	M2
*OSBP2*	1q	M2
*SLITRK5*	1q	M2p
*ABCA12*	3p	2q (1 homologue)
*G6PD*	Xp	M1p
*TMHLE*	Xp	M1p
*MECP2*	Xq	M4p
**Copy Number Increase**		
*OCLN*	1p	M1, M3, 2p (1 homologue)
*IPO8*	5p	3q, 5p
*BET1L*	6p	6p, 6q, M3q

## Discussion

Since the first characterisation of DFTD tumour chromosomes by G-banding [8], there has been avid interest in analysis of DFTD at the genome level. The identification of genomic rearrangements on a genome-wide scale in human cancers has advanced in recent years from low resolution karyotyping techniques such as G-banding and spectral karyotyping (chromosome painting), to more sensitive techniques such as array genotyping and approaches employing next generation sequencing capable of identify all types of mutations including SNPs, small insertions-deletions, copy number variants and translocations [21], and providing resolution of breakpoint rearrangements at the base pair level [22]. Without access to a high quality anchored reference genome sequence, this type of detailed tumour analysis is challenging for other species, particularly for the devil, a species for which no studies at the genome-level have been previously performed.

Here, by using a combination of chromosome painting and gene mapping we provide the first details of the extent of rearrangement in the tumour and the origin of the four marker chromosomes. Our mapping data provides a framework for an anchored assembly of genome sequence data of the normal devil and tumour genomes, an essential resource required for sensitive tumour genome sequencing approaches to identify all types of mutations within tumours.

The complementary approaches of chromosome painting and gene mapping provided much better resolution of tumour genome rearrangement than either approach alone. Chromosome painting revealed large regions of homology between normal and tumour karyotypes and enabled differences between the tumour strains to be identified at this gross level. Gene mapping enabled rearrangements within these regions of homology to be detected and provided detail of the regions of the genome that are rearranged, increased in copy number or deleted in tumour cells. The extent of rearrangement we discovered in the three different tumour strains we investigated warrants a more detailed gene map to completely cover each of the tumour strains. In the absence of a detailed map or assembled sequence, chromosome painting identifies regions of homology in gaps, and regions poorly covered, such as chromosome 5. Comparisons with maps for other marsupial species, and even humans, provide null hypotheses for the gene content of the tumour chromosomes and predict the genes close to tumour breakpoints.

### Candidate genes involved in tumourigenesis

The transformation of a normal cell to a cancerous one involves the accumulation of mutations, often in tumour suppressor genes or oncogenes. There is a growing list of such genes perturbed in human cancers, making it difficult to know where to begin searching for candidate genes involved in tumourigenesis in DFTD.

**Figure 8:**
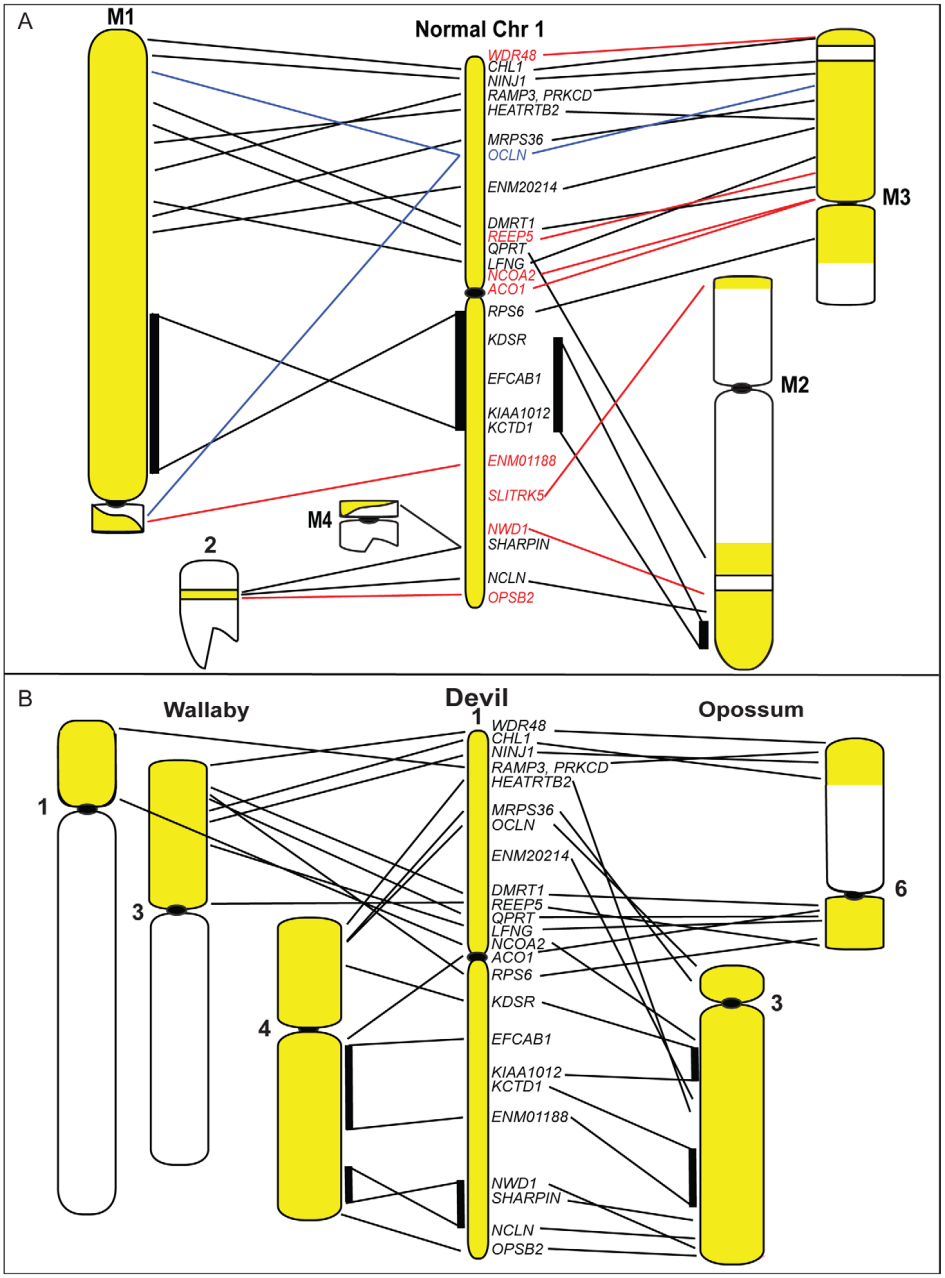
Chromosome 1 rearrangements in DFTD and during marsupial evolution. (A) A comparison of gene arrangement on the normal devil chromosome 1 to arrangement observed on DFTD chromosomes shows extensive rearrangement, with only one block of genes (*KDSR* to *KCTD1*) conserved in gene order between normal chromosome 1 and DFTD. Yellow regions on DFTD chromosomes indicate homology to normal devil chromosome 1. Genes shown in red mapped to only one location in DFTD, whereas the gene in blue mapped to three different locations. (B) Comparison of gene order of devil chromosome 1 with arrangement in wallaby (left) and opossum (right).

Our mapping data allows us to predict where many of the most common tumour suppressor genes and oncogenes are located in DFTD, and whether these sites are located in regions of the devil genome that were rearranged in the tumour. From the list of common cancer genes (Table 3), we find that a large number are located on devil chromosome 1. Significantly, this chromosome has undergone extensive rearrangement in the tumour (Figure 8A). Several genes (*APC, MYC, NF2, MLH1*) stand out as potentially playing a role in DFTD tumourigenesis, being predicted to be close to genes that have one copy deleted in DFTD (*REEP5*, *ENM01188*, *OSBP2*, *WDR48* respectively) and hence, they themselves may be perturbed.

**Table 3:** Predicted location of common tumour suppressor genes and oncogenes in the devil genome.

Gene	Opossum Chromosome	Predicted Devil Chromosome	Nearest Mapped Gene	Distance from mapped gene (Mb)
*BCL2*	3	1	*KDSR*	0.2
*MYC*	3	1	*ENM01188*	8
*NF2*	3	1	*OSBP2*	2
*APC*	6	1	*REEP5*	0.07
*MLH1*	6	1	*WDR48*	0.7
*PTEN*	1	2	*MINPP1*	0.3
*BRCA2*	4	3	*SPG20*	5
*RB1*	4	3	*FREM2*	8
*MYCL1*	4	3	*CCKBR*	9
*RAD50*	5	3	*KLHL1*	2 and 9
*BRCA1*	2	4	*SOST*	2
*ERBB2*	2	4	*SOST*	6
*TP53*	2	4	*PGBD2* & *C17orf101*	6 and 10
*NF1*	2	4	*TPST1*	4

Predictions of location in the normal devil genome are based homologies revealed by cross-species chromosome painting. Distance of cancer genes from mapped genes is based on the distance in opossum genome assembly. Only those cancer genes located within a 10 Mb interval either side of a mapped gene are listed.

The Schwann cell origin of DFTD [7] makes the tumour suppressor *NF2* a particularly interesting gene to examine more closely in future studies. In humans, loss of *NF2* function is linked to tumours of the central nervous system, particularly benign tumours such as schwannomas [23], although in mice loss of *NF2* has been associated with a variety of malignant tumours [24]. We predict, based on the opossum genome assembly, that *NF2* is approximately 2 Mb away from the mapped gene *OSBP2*, a gene that maps to only one position on the short arm of chromosome 2 in DFTD (Figure 8A).

### DFTD evolution

Devil facial tumour disease is a rare exception to established models of tumour development and progression, as demonstrated by cytogenetic evidence [8].

The classic model of stepwise carcinogenesis describes a gradual process in which neoplastic cells progress through a spectrum of increasingly malignant phenotypic changes that correlate with escalating genomic chaos [25]. This is best exemplified by human colorectal tumours, in which the transformation of benign dysplastic lesions into invasive carcinomas is associated with an accumulation of gross cytogenetic aberrations [26], [27]. Randomly acquired genetic mutations that afford neoplastic cells a competitive advantage are propagated in waves of clonal expansion so that increasingly malignant cells are selected for in a process akin to Darwinian evolution. By contrast, the cancer stem cell (CSC) model posits that only a proportion of neoplastic cells have the capacity for self-renewal and tumour initiation, and these cells are the drivers of malignancy [28]. These two theories are neither conflicting nor mutually exclusive, and both account for the intra-tumoral heterogeneity typically present in solid and hematologic malignancies.

In contrast, DFTD is a stable, clonal cell line transmitted from animal to animal by biting. Its biological behaviour within wild devil populations renders it a somatic cell pathogen that forms proliferative masses upon transplantation. A lack of genetic diversity between animals at functionally important MHC loci [9] and the epidemiologic dynamics of DFTD transmission [29] set the stage for the devastating disease outbreak that now threatens extinction of Tasmanian devils. The genomic events that underpinned the formation of the original devil tumour are uncertain; however, our chromosome painting and BAC mapping results have pinpointed candidate genes and elucidated the gross cytogenetic restructuring that produced the original tumour and switched a Schwann cell in a single sentinel animal into the pathway to carcinogenesis.

### Origin and evolution of DFTD

Consistent with previous G-banding and genotyping results [7], [8], [9] our chromosome painting experiments support the hypothesis that DFTD derived from a clonal cell line in 1996. The absence of Y-chromosome sequences (Figure S5) suggests that the sentinel animal that harboured the original tumour was female. The presence of two copies of 11 out of 14 X-borne genes supports this hypothesis.

It is possible that the neoplastic cell that ultimately became transmissible was a clonal stem cell (CSC). This is consistent with the limited heterogenetiy of neoplastic cells, their poorly differentiated morphology [30] and their gene expression profile [7]. Our observation of limited divergence into several strains and sub-strains implies that the basal tumour karyotype was established early in tumour evolution, and has remained extraordinarily stable over the subsequent fifteen years. Thus an alternative hypothesis is that all tumour strains are the same age and represent various subclones of an original, heterogenous tumour in the sentinel animal. However, subclones must have been all capable of self-renewal and tumour initiation, which seems rather unlikely as few cells independently acquire properties of CSCs.

A third, intriguing, possibility is that the DFTD karyotype was generated in a single episode of massive genomic restructuring. Termed chromothripsis, this phenomenon was recently described in a variety of solid and hematologic malignancies [31]. The genomic signature of chromothripsis is typified by complex remodelling of a small number of chromosomes with minimal loss of heterozygosity and variation in gene copy number. It is clear that complex chromosome rearrangements in DFTD are localised to well demarcated genomic regions. BAC mapping results demonstrate that chromosomes 1 and X are particularly fragmented, with dozens of DNA breaks and fusions contained to only a small portion of the genome. Our observation that chromosome 1 has undergone the same numerous rearrangements in all strains suggests that rearrangement of this chromosome as a result of chromothripsis was the initial step in the development of DFTD.

Stephens *et al* [31] suggest that chromothripsis occurs when cells undergo catastrophic chromosome rearrangements, during which well delineated regions of the genome are reduced to tens or hundreds of fragments that are haphazardly fused by nonhomologous end-joining DNA repair machinery. What might incite such dramatic genomic restructuring is unknown, though the authors suggest that breakage-fusion-bridge (BFB) cycles associated with telomere loss could cause the catastrophic genomic restructuring of chromothripsis. This is a particularly intriguing speculation, as telomere length varies between chromosomes; those chromosomes with the shortest telomeres are predisposed to telomeric fusions and are consequently drivers of BFB cycles and chromosome rearrangement [32]. The DFTD karyotype may be a snapshot of a brief period of localised genomic instability associated with focal telomere attrition, eventually rescued by recruitment of telomerase expression.

### The DFTD karyotype is clonal and stable

The clonal passaging of DFTD from animal to animal over a protracted period provides a unique opportunity to study the long-term karyotype evolution of a solid tumour.

Surprisingly, we found that cytogenetic differences between tumour strains are minimal. The eight DFTD cell lines examined in this study were established from primary lesions in male and female devils trapped in various locations throughout Tasmania over a period of three years (Figure S2). We found both inter-strain and intra-strain differences of similar magnitude, highlighting the stability of the DFTD genome while suggesting that karyotype evolution continues. Additionally, the presence of multiple sub-strains suggests that upon transmission, the tumour inoculum contains mixtures of cell lines that may have diverged over some years. For instance, the two 3B sub-strains are distinguished by the variable loss of marker chromosome M5, subtle variations in chromosome 5 rearrangements and the absence of an additional chromosome 4 rearrangement that marks other Strain 3 tumours. The differences within this tumour are more complex than the subtle rearrangements that distinguish Strains 1 and 2. This observed pattern of intra-tumour chromosome variability is consistent with observations that the tumour is passed from animal to animal by biting, during which many clumps of tumour cells are dislodged from the mouth of the affected animal [33].

The long-term stability of tumour chromosomes, both *in vivo* and *in vitro*, indicates that DFTD does not share the overt genomic instability typical of many solid tumours in humans and mice. Nevertheless, the predominance of chromosome 4, 5 and X permutations among and within strains may correlate with mild chromosome instability localised to these chromosomes. Perhaps selection is acting on the DFTD karyotype to maintain the tumourigenic properties of a DFTD cell, while tolerating genomic instability in regions of the genome not essential for survival of a DFTD cell. This is consistent with the hypothesis that chromosome 1 rearrangement was the initial step in the development of DFTD and that the maintenance of these rearranged chromosome 1 regions is critical for the survival of DFTD in the devil population. Conversely, continued perturbations of chromosomes 4, 5 and X are neutral, having no affect on DFTD tumourigenesis.

There are no data that attaches any clinical significance to the karyotypic strains, nor is it known whether the emergence of new karyotypic strains correlates with meaningful phenotypic changes. The provision of detailed descriptions of strain karyotypes will make it possible to investigate this important question in more depth.

### Are devil tumour breakpoints associated with marsupial evolutionary breakpoints?

It appears that certain regions of the human genome are ‘hotspots’ for rearrangement in tumours [34] and there has been much debate about whether these regions are the same parts of the genome that display the most rearrangement when comparisons of gene arrangement are made between eutherian mammals. Cancer-associated breakpoints in humans have been frequently reported to co-localise with evolutionary breakpoints, regions in which chromosomal breaks have occurred more than once during eutherian evolution [34]. However, a more recent study which localised breakpoints on a much finer scale refuted this claim by finding no evidence of more frequent co-localisation of evolutionary and cancer breakpoints [35]. Perhaps evolutionary and tumour breakpoints do not occur at exactly the same base pair position in the genome, but are concentrated in specific regions of the genome that are more susceptible to breakage, both during the course of evolution, and tumourigenesis.

Intriguingly, the chromosomes most rearranged in DFTD tumour lines are the same ones that are most rearranged between devil, wallaby and opossum genomes. Chromosome 1 is a good example, since there has been extensive rearrangement of this chromosome in DFTD and between different marsupial species (Figure 8). Furthermore, the same parts of this chromosome are less or more subject to rearrangement both in the tumour and between species. The region from *EFCAB1* to *KCTD1* on the long arm of chromosome 1 is intact (conserved in gene order) on both marker chromosomes M1 and M2, and is conserved (gene order) as a block in wallaby and devil, suggesting that this region has been less susceptible to rearrangement in DFTD and during marsupial evolution. The remainder of chromosome 1 is highly rearranged in DFTD, being spread across five chromosomes and with eight out of 12 genes present in only a single copy and one gene mapping to three different locations (Figure 8A). This region has undergone extensive reshuffling between devil, wallaby and opossum (Figure 8B). Regions of the genome that are relatively well conserved between species (e.g. the long arm of devil chromosome 3, see Figure 2A) have remained unchanged in DFTD. Genome sequence data is required to determine whether there are sequence features in common between regions susceptible to rearrangement.

### Conclusions

The emergence of DFTD has had a disastrous effect on wild Tasmanian devil numbers, and with the devil now perilously close to extinction, intense research efforts to understand and intercept DFTD pathogenesis proceed apace. Here we contribute a detailed map of the global chromosome restructuring and intricate gene rearrangements that characterise DFTD. We provide further confirmation of the clonal transmission of DFTD and tentatively identify the sentinel animal as a female devil. Our observation that only limited regions of the genome are highly rearranged suggest that chromothripsis was the mechanism of the original tumorigenesis, and, once remodelled, the tumour karyotype has been remarkably stable during its clonal transmission from animal to animal.

By anchoring genes to a reference and tumour maps, we can predict the locations of common tumour suppressor genes and oncogenes. By characterising multiple strains and sub-strains we have demonstrated the stability of the tumour genome. This study provides an important framework for future genomic studies into DFTD.

## Materials and Methods

### Ethics statement

The collection of samples from devils was approved by the Australian National University Animal Experimentation Ethics Committee (AEECP R.CG.11.06).

### Sample collection and tissue culture

Tissue samples for tumour culture were obtained from biopsies of live, wild caught Tasmanian devils and from necropsy specimens. Wild Tasmanian devils were trapped for the purposes of disease surveillance and epidemiologic studies, and were biopsied under general anaesthesia. DFTD-affected animals that were euthanased, either for humane reasons or because they were trapped in disease exclusion sites [36], were necropsied in the field or at the Tasmanian Animal Health Laboratory. Samples were sourced from a variety of geographic locations in order to obtain representative cultures of each of the three tumour strains. Primary tumour cultures were initiated according to the Pearse and Swift [8] protocol. Briefly, tumour biopsies were washed in 10 mL Dulbecco's phosphate buffered saline (PBS) (Invitrogen, Mulgrave, VIC, Australia) supplemented with 0.1 mL penicillin-streptomycin solution (Sigma-Aldrich, Castle Hill, NSW, Australia). Cultures were established by manually disaggregating tumour tissue using a scalpel, followed by re-suspension in 8 mL GIBCO AmnioMAX-C100 (Invitrogen). Cultures were incubated at 35°C in 5% CO_2_ and harvested after 24 to 48 hours for diagnostic purposes and to ensure that additional chromosome rearrangements did not occur in subsequent passages.

### Metaphase chromosome preparation

Metaphase chromosomes were prepared from a normal male devil cell line (passage 3) and DFTD cultures according to standard techniques [8]. In brief, cultures were harvested after a 2 hour synchronisation with colcemid (10 µg/mL) by incubating in 37°C, hypotonic solution (0.075 mM KCL) for 18 minutes and fixation with chilled methanol∶acetic acid (3∶1). Cell suspensions were dropped on to slides, air-dried and stored for 24 hours prior to hybridisation.

### Chromosome painting

A panel of six chromosome paints comprising all autosomes and the X chromosome were hybridised to metaphase chromosomes from each of the three tumour strains. Chromosome paints for devil chromosomes 1 to 6 and the X were generated from flow sorted *S. harrisii* chromosomes as previously described [37]. The Y chromosome paint was produced by manual microdissection of metaphase chromosomes, freshly dropped onto glass coverslips and collected with a glass needle mounted on a Ziess Axiovert I microscope [38]. Primary degenerate oligo-primed (DOP) PCR products were labelled with biotin-dUTP or digoxygenin-dUTP (Roche Diagnostics, Basel, Switzerland) in subsequent amplifications by DOP-PCR with 6MW primer (5′-CCG ACT CGA GNN NNN NAT GTG G-3′) [39]. The labelled PCR product was co-precipitated with C_o_t-1 DNA (5 ug/slide) for suppression [40], suspended in 15 µl of pre-warmed hybridisation buffer (50% formamide, 2× SSC, 10% dextran sulfate) and denatured at 70°C for 10 min and pre-annealed for 20 min at 37°C. Metaphase spreads were denatured for 40 seconds in a 70% formamide solution at 70°C and hybridised overnight at 37°C. Post hydridisation washes were performed according to Alsop et al [40]. Biotin and digoxygenin-labelled probes were detected with avidin-FITC (Vector Laboratories Inc., Burlingame, CA, USA) and anti-digoxygenin-Cy3 (Roche Diagnostics), respectively. DAPI (4′,6-diamidino-2-phenylindole) was used as a counterstain and slides were mounted in Vectashield (Vector Laboratories Inc., Burlingame, CA, USA). A Zeiss Axioplan2 epifluorescence microscope was used to visualise fluorescent signals which were captured with a SPOT RT Monochrome charged-couple device camera (Diagnostic Instruments Inc., Sterling Heights, MI, USA) and processed using IP Lab imaging software (Scanalytics Inc, Fairfax, VA, USA).

### BAC library construction

A bacterial artificial chromosome (BAC) library, designated VMRC-50, was produced using the detailed procedures of library construction described previously [41], [42]. This library was constructed from genomic DNA extracted from the liver of a deceased two-year-old male devil (Accession Number 08/0134) that was originally from Bangor, Tasmania and euthanized in 2008 due to multiple DFTD lesions and metastases to the lungs. Quality of the DNA was checked by running a pulsed field gel electrophoresis (PFGE) on a CHEF -DR III system (BioRad, Hercules, CA, USA). The DNA was partially digested in an *EcoRI/EcoRI-methylase* competition reaction and size fractionated by analytical PFGE on a CHEF Mapper XA system (BioRad). DNA fragments from the appropriate size fraction were ligated into the CopyControl pCC1BAC vector from Epicentre Technologies and transformed into ElectroMAX DH10B T1 Phage-Resistant *E. coli* cells (Invitrogen). Transformants were arrayed into 384-well LB/chloramphenicol/glycerin microtiter plates (Genetix, San Jose, CA, USA) using colony-picking robot (Norgren Systems, Fairlea, WV, USA) and subsequently gridded onto 22×22 cm high-density nylon filters with a Total Array System (BioRobotics Ltd., Woburn, MA, USA).

### Overgo design and BAC library screening

Genes located near the ends of opossum-wallaby conserved gene blocks were identified by comparing the arrangement of genes between the anchored opossum genome sequence [16] and physical map of the wallaby genome (Deakin et al, in preparation; [43], [44]). Opossum orthologues for genes located near the ends of these blocks were found in the Ensembl gene build (MonDom5) and used to search the available devil transcriptome sequence [7] with BLASTN. Devil-specific overgos were designed using the Overgo Maker program (http://genome.wustl.edu/software/overgo_maker) using the devil orthologous sequence as the input sequence. Specificity of the resulting 40 bp probe was confirmed by BLASTN searches of the devil transcriptome, as well as the wallaby and opossum genome assemblies. Proposed overgos matching numerous positions in the wallaby and opossum genomes or many contigs in the devil transcriptome were discarded in order to avoid the detection of paralogous genes. A complete list of the overgos used in this study is provided (Table S1). BAC library filters were screened with pools of up to 60 radioactively labelled overgo pairs using the protocol described by Ross et al [45]. Dot blots were performed as described by Deakin et al [43] on the resulting positive BACs in order to determine which BACs were positive for each gene. BACs mapping to different chromosomes than predicted were subjected to direct sequencing, using an overgo as a sequencing primer according to the previously described protocol [43].

### Fluorescence in situ hybridisation (FISH)

DNA from each BAC clone was isolated using the WIZARD Plus SV Minipreps DNA Purification System (Promega, Alexandria, NSW, Australia), and approximately 1 µg of DNA was labelled by nick translation with either SpectrumOrange dUTP or SpectrumGreen dUTP (Abbott Molecular Inc., Des Plaines, IL, USA). Labelled probes were hybridised overnight to normal male devil or DFTD tumour metaphase chromosomes following the protocol detailed in Alsop et al [40] with one exception. Denaturation time for normal male chromosomes was 1 min 40 but, as the tumour chromosomes were observed to be more susceptible to overdenaturing, the denaturing time was reduced to 1 min for DFTD tumour chromosomes. Unbound probe was washed off slides with one wash of 0.4×SSC with 0.3% (v/v) Tween 20 for 2 min at 60°C, followed by a wash at room temperature in 2×SSC with 0.1% (v/v) Tween 20 for 5 sec to 1 min. Chromosomes were counterstained in DAPI and mounted with Vectashield (Vector Laboratories Inc.). Fluorescent signals were visualised using a Zeiss Axioplan2 epifluorescence microscope. Images of both DAPI stained chromosomes and fluorescent signals were captured on a SPOT RT Monochrome CCD charge-coupled device camera (Diagnostic Instruments Inc.) and merged using IP Lab imaging software (Scanalytics Inc).

## Supporting Information

Figure S1 Sequence alignments for BACs mapping to unexpected location. Sequences obtained from BACs using overgos as sequence primers are aligned to either coding sequence or conserved intronic sequence from opossum orthologues to show that mapped BACs to contain the relevant genes.
(PDF)

Figure S2 Comparison of gene arrangement between devil, wallaby and opossum chromosomes. (A) Gene order for the grey shaded region on devil chromosome 3 is considerably rearranged between species. (B) Devil chromosome 2 has large regions conserved in gene order between wallaby and opossum. (C) Devil chromosome 4 has a few blocks of genes conserved in gene order between wallaby and opossum. Opossum chromosome 2 has been inverted to make it easier to illustrate the conserved gene blocks.
(TIF)

Figure S3 Flow karyotype of *Sarcophilus harrisii*.
(PDF)

Figure S4 Information on Strains used in this study. The locations of where samples for each strain were collected are indicated on the map of Tasmania. Additional information, such as the sex and chromosome paints used on each sample, is indicated in the table below the map.
(PDF)

Figure S5 Chromosome painting using the microdissected Y chromosome on normal and DFTD chromosomes. A DAPI stained image of the chromosomes is shown on the left and hybridisation with the Y chromosome paint on the right. A clear hybridisation signal is evident on the Y chromosome on the normal male metaphase spread but not on DFTD chromosomes.
(TIF)

Figure S6 A summary of the chromosome painting differences between the three different Strain 3s. Differences between Strains 3A, 3B and 3C were detected with paints for chromosomes 4, 5 and X, and substrains of 3B and 3C were observed.
(PDF)

Figure S7 Images of the chromosome 4 and 5 paints on metaphase spreads from a normal female and DFTD tumour strain 3.
(PDF)

Figure S8 A comparison of gene arrangement on the normal devil chromosomes with arrangement observed on DFTD Strain 1 chromosomes 2 (A), 3 (B), 4 (C), 5 (D), 6 (E) and X (F). Genes in red are present in only one copy in DFTD and genes in blue are present in 3 copies.
(PDF)

Table S1 List of genes mapped, the overgos used for library screening and the BACs positive for each gene.
(XLS)

Table S2 Success rate of overgos used for library screening.
(DOCX)
