# Correction: Genomic Restructuring in the Tasmanian Devil Facial Tumour: Chromosome Painting and Gene Mapping Provide Clues to Evolution of a Transmissible Tumour

**DOI:** 10.1371/journal.pgen.1004840

**Published:** 2014-10-31

**Authors:** The PLOS Genetics Staff

An error was made in record keeping at the very start of this study regarding the strain classification of four of the tumour samples. This error affects sections in the Results and Discussion sections, along with Figures 4, 7, S4, S6 and S7. Overall, references to strain 2B should refer to strain 3A, strain 3A to strain 2B, strain 3B to 2C and strain 3D to 2D. Strain 3C has been renamed 3B since there are now only two strain 3 samples in this study.

**The 5^th^, 6^th^ and 7^th^ paragraphs of the section entitled ‘Chromosome painting on DFTD tumour cell line strains’ are incorrect. The correct paragraphs are:**

Based on both G-banding and chromosome painting results, strain 1 cells were found to retain the basic DFTD karyotypic framework, whereas Strains 2 and 3 were marked by additional rearrangements. In most Strain 2 and Strain 3 tumours, an additional marker chromosome M4 was hybridized by the chromosome 4 paint throughout the long arm, and an additional reciprocal translocation between chromosomes 4 and 5 (Figures S6 and S7). These strains had an additional marker chromosome M5, which completely hybridised to the X paint (Figure 4, Figure S6).

Strain 2 and 3 karyotypes were found to be somewhat more complicated than Strain 1, showing variation of painting patterns between tumour cell lines isolated from different animals, and the presence of two distinct sub-strains in two tumours examined. M4 was variably present in Strain 2 and 3 tumours, with loss of this marker in 0–64% of metaphases in different tumour cell lines (see Figure S6). The variable loss of M4 was interpreted as a relatively minor change and was not considered indicative of more broad scale karyotypic instability. An additional translocation between chromosomes 4p and M4q was present in some cells in Strains 2B and 3B. This translocation was present in all metaphases of Strain 2B, compared with only 12.5% (1 out of 8) of Strain 3B metaphases, and was absent in Strain 2C. Strain 2C also exhibited some heterogeneity; 36% of cells lacked M5 (18 out of 50), and 58% (29 out of 50) lacked M4. In cells lacking M4, the chromosome 5 paint hybridised to the short arm of the giant marker, replacing the X chromosome signal present at this location in all other tumours. In the 36% (18/50) of tumours that had M4, the chromosome 5 paint hybridised to the long arm of M4, as for Strain 1 tumours.

Paints generated from flow-sorted normal devil chromosomes have therefore revealed the origin of the genomic material that comprises each marker chromosome, as well as several insertions undetectable with G-banding. Painting also demonstrated the extent to which chromosomes 1, 4, 5 and the X chromosome are rearranged in DFTD. None of this information could be gained from earlier G-banding studies. Our findings indicate that progressive rearrangements of chromosomes 4, 5 and the X chromosome distinguish Strain 1 from Strains 2 and 3, and that some Strain 2 and 3 tumours are composed of at least two sub-strains, present in varying proportions, implying that passage of the tumour from animal to animal is usually via multiple cells.

**The 6^th^ and 7^th^ paragraphs of the section entitled ‘Physical map of DFTD tumour cell strains’ are incorrect. The correct paragraphs are:**

A readily distinguishable difference between G-banded karyotypes of tumour strains was found to be the deletion of part of the short arm of chromosome 3 uniquely in Strain 3. We have confirmed this by gene mapping and show that the region deleted spans from *MDH1B* on distal 3p to *TGFBRAP1* on proximal 3p but the deletion was only detected in one of the two Strain 3 samples, as well as in Strain 2. Only one copy of the chromosome has this deletion in Strains 2C and 3C, but both copies have the deletion in Strain 2B (Figure 7) and no signals were observed for these genes on any other chromosome, suggesting these genes are completely absent from the tumour genome. The deletions appeared to be the same on both copies of chromosome 3, suggesting that the normal member of the pair may have been lost, and the deleted copy reduplicated.

The Strain 2 and 3 tumours also have variations in the arrangement of chromosome 4 and 5 genes (Figure 7). Genes from the short arm of chromosome 4 were observed to be absent from one copy of the chromosome in Strain 2B, and this deletion is also present in 20% of Strain 3B metaphase spreads. In addition, Strain 2C was found to have retained *TPST1*and *SENP2* on chromosome 4 (these genes were found on M4 in all other strains), although *SENP2* was observed to be translocated to 4q. This strain was shown also to have acquired an additional copy of *C17orf101* on the short arm of M2. Strain 3B had three copies of *ST6GALNAC5*, one copy on each of the chromosome 4 homologues observed in all strains, as well as an additional copy on the short arm of M2. In most Strains 2 and 3, chromosome 5 genes were detected on the short arm of M2 and M5 (except 3A).

**The 2^nd^ paragraph of the section entitled ‘The DFTD karyotype is clonal and stable’ is incorrect. The correct paragraph is:**

Surprisingly, we found that cytogenetic differences between tumour strains are minimal. The eight DFTD cell lines examined in this study were established from primary lesions in male and female devils trapped in various locations throughout Tasmania over a period of three years (Figure S4). We found both inter-strain and intra-strain differences of similar magnitude, highlighting the stability of the DFTD genome while suggesting that karyotype evolution continues. Additionally, the presence of multiple sub-strains suggests that upon transmission, the tumour inoculum contains mixtures of cell lines that may have diverged over some years. For instance, the two 2C sub-strains are distinguished by the variable loss of marker chromosome M5 and subtle variations in chromosome 5 rearrangements. The differences within this tumour are more complex than the subtle rearrangements that distinguish Strain 1 from Strains 2 and 3. This observed pattern of intra-tumour chromosome variability is consistent with observations that the tumour is passed from animal to animal by biting, during which many clumps of tumour cells are dislodged from the mouth of the affected animal [33].

**Figure 4** **and** **Figure 7** **are incorrect, as are their legends. Corrected versions are provided here.**

**Figure 4:**
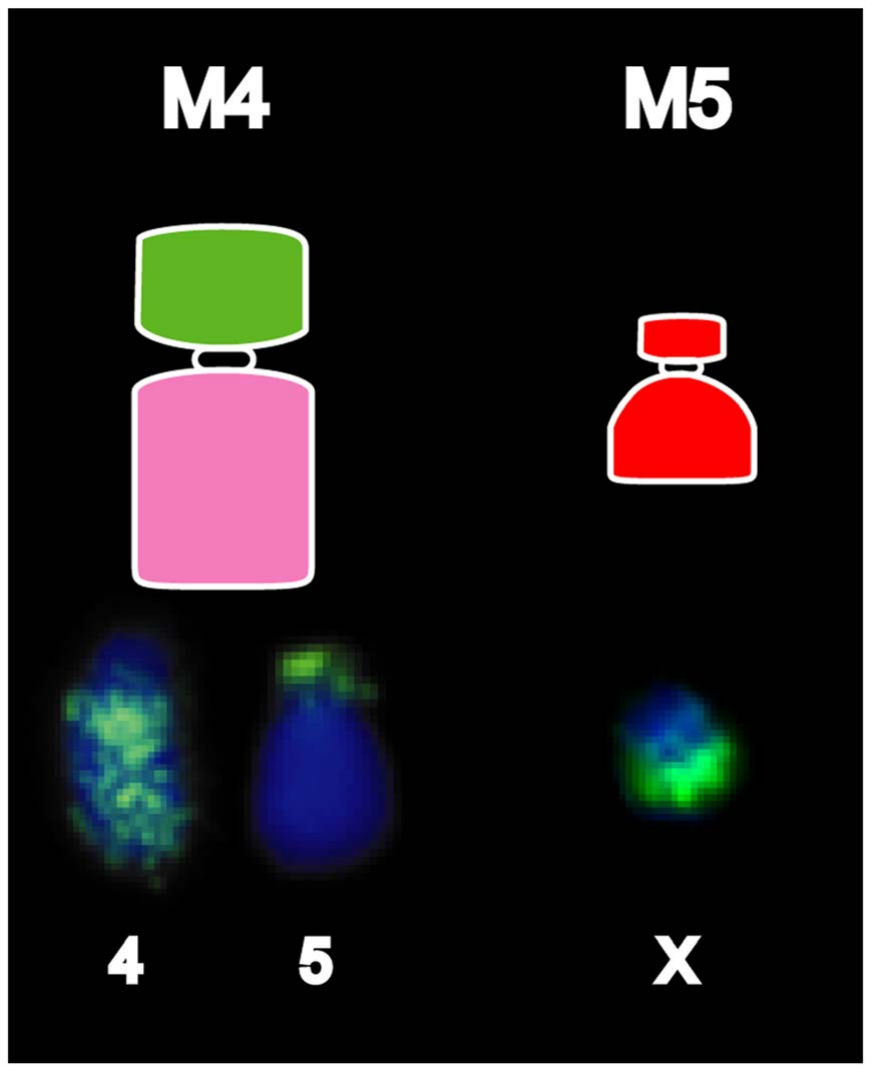
Chromosome painting results unique to DFTD strain 2. Differences detected between Strain 1 and Strains 2 and 3 typically involve the detection of chromosome 4 on M4 and X chromosome on an additional marker chromosome, M5.

**Figure 7:**
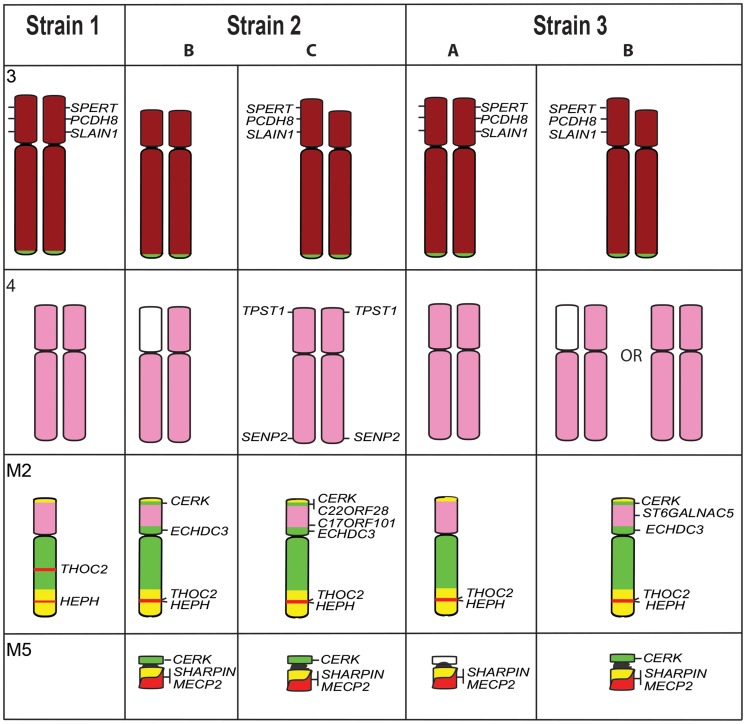
Differences detected by gene mapping among Strains 1, 2, and 3. Genes *SPERT*, *PCDH8* and *SLAIN1* are found on both homologues of chromosome 3 in Strains 1 and 3A (gene names are only indicated next to one homologue) but a deletion of these genes has occurred on both homologues of Strain 2B, and one homologue of Strain 2C and 3B. Chromosome 4 is different among the Strains 2 and 3. In Strain 2B, genes mapped only to the short arm of one copy of chromosome 4 Strain 2C has retained *TPST1* on 4p, a gene mapping to M2 and M4 in all other strains, and the 4p *SENP2* gene, has translocated to 4q. X chromosome genes *THOC2* and *HEPH* map to different location on M2 in Strain 1 but colocalise in other strains. Strains 2 and 3 have an additional marker chromosome (M5), which contains *SHARPIN* and *MECP2* in Strain 2 and 3, as well as *CERK* in Strain 3. Colour coding of chromosomes is the same as that used in Figure 3.

**Supplementary Figure S4 is incorrect. Supplementary Figure S6 and Supplementary Figure S7 are incorrect, as are their legends. Corrected versions are provided here.**

## Supporting Information

Figure S4 Information on Strains used in this study. The locations of where samples for each strain were collected are indicated on the map of Tasmania. Additional information, such as the sex and chromosome paints used on each sample, is indicated in the table below the map.
(JPG)

Figure S6 A summary of the chromosome painting differences between strains (in addition to those depicted in Figure 4). Differences between Strains 2B, 2C and 3B were detected with paints for chromosomes 4, 5 and X, and substrains of 2C and 3B were observed.
(JPG)

Figure S7 Images of the chromosome 4 and 5 paints on metaphase spreads from a normal female and DFTD tumour strain 2.
(JPG)
