# Supplementary material for: Genomic Restructuring in the Tasmanian Devil Facial Tumour: Chromosome Painting and Gene Mapping Provide Clues to Evolution of a Transmissible Tumour
Source: PLoS Genet. 2012 Feb 16;8(2):e1002483. doi: 10.1371/journal.pgen.1002483 (PMC3280961; doi:10.1371/journal.pgen.1002483)
Supplement: Figure S1 — Sequence alignments for BACs mapping to unexpected location. Sequences obtained from BACs using overgos as sequence primers are aligned to either coding sequence or conserved intronic sequence from opossum orthologues to show that mapped BACs to contain the relevant genes. (PDF) [file pgen.1002483.s001.pdf]

A) Alignment of *ARL4A* sequence (coding sequence)

```
devil           GACGCTGGGCAACGCCAAGACGGTGACGTTCCACTTCTGGGACGTGGGCGGCCAGGAGAA 60
opossum         GACGCTGGGCAACGCCAAGACTGTGACCTTCCACTTTTGGGACGTGGGCGGCCAGGAGAA 60
                ********************* ***** ******** ***********************

devil           GCTGCGGCCGCTCTGGAAGTCGTACACGCGCTGCACCGACGGCATCGTGTTCGTGGTGGA 120
opossum         ACTGCGGCCGCTCTGGAAGTCGTACACTCGCTGCACCGACGGCATCGTCTTCGTCGTGGA 120
                .************************** ******************** ***** *****

devil           CTCGGTGGACGTGGAGCGCATGGACGA 147
opossum         CTCTGTGGACGTGGAGCGCATGGACGA 147
                *** ***********************
```

B) Alignment of *BET1L* sequence (coding sequence)

```
devil           GGACTCGGACTTGTCCAGCGTGACCAGCCTGCTGAGCGGGAGCGTGAAGCGCTTCTCCAG 60
opossum         GGACTCGGACTTCTCCAGCGTGACCAGCCTGCTGAGCGGGAGCGTGAAGCGCTTCTCCAC 60
                ************ ***********************************************

devil           TCTGAGCCGCTCGGGCCGGGACAACCGGCGGCTGCTGCTCTGCGTGTCTGTGGGACTCAT 120
opossum         CATGACCCGCTCAGGCCGTGACAACCGGCGGCTGCTGCTCTGCGTGGCTGTGGGGCTCAT 120
                 .*** ******.***** *************************** *******.*****

devil           C 121
opossum         C 121
                *
```

C) Alignment of *LECT1* sequence (conserved intron sequence)

```
devil           TCCAGATAATCCTTACCATGTGAG---------TCACATTAATTCAGCACTAAAATGATG 51
opossum         TCCAGATAATCCTTATCATGTGAGTAGTCATGTTCAGATTAACACAGCTCTAAAATAATG 60
                *************** ********         *** *****  :****:*******.***

devil           TTTCAGCATATTTACTTTAAAATAAACATTTAACATTCAATGGAAACTTTTTTGTGATTT 111
opossum         TTTCTGCATATGTATTTTGAAACAAGTATTTAACACTCAATGGAAATGTTTTTCTGATTT 120
                ****:****** ** ***.*** **. ******** **********  ***** ******

devil           AGTAATAACTT 122
opossum         AGTAATAACTT 131
                ***********
```

D) Alignment of *SLITRK5* sequence (coding sequence)

```
devil           AATCAGCCCTCCACGATTTCCAGTCTACCACCTCTTGTTGTCTGGCAACCTTCTGAACCG 60
opossum         AATCAGCCCGCCACGCTTTCCAGTCTACCACCTCTTGTTGTCTGGCAACCTTTTGAACCG 60
                ********* *****.************************************ *******

devil           TCTCTACCCCAATGAGTTTGTCAATTACACTGGGGCTTCAATTTTACATCTAGGGAGCAA 120
opossum         TCTCTACCCTAATGAGTTTGTCAATTACACTGGGGCTTCAATTTTACATTTAGGGAGCAA 120
                ********* *************************************** **********

devil           TGTCATCCAGGACATTGAGACAGGGGCATTTCATGGGCTTCGAGGTTTAAGGCGATTGCA 180
opossum         TGTCATCCAGGACATTGAGACAGGGGCATTTCATGGGCTTCGAGGTTTGAGGCGATTACA 180
                ************************************************.********.**

devil           TCTGAATAATAATAAACTGGAACTACTAAGGGATGACACTTTCCTTGGATTGGAGAGTCT 240
opossum         TCTGAATAATAATAAACTGGAATTACTAAGGGATGACACTTTCCTTGGATTAGAGAGTCT 240
                ********************** ****************************.********

devil           GGA 243
opossum         GGA 243
                ***
```
