# Supplementary figures and images for: Genomic Restructuring in the Tasmanian Devil Facial Tumour: Chromosome Painting and Gene Mapping Provide Clues to Evolution of a Transmissible Tumour
Source: PLoS Genet. 2012 Feb 16;8(2):e1002483. doi: 10.1371/journal.pgen.1002483 (PMC3280961; doi:10.1371/journal.pgen.1002483)

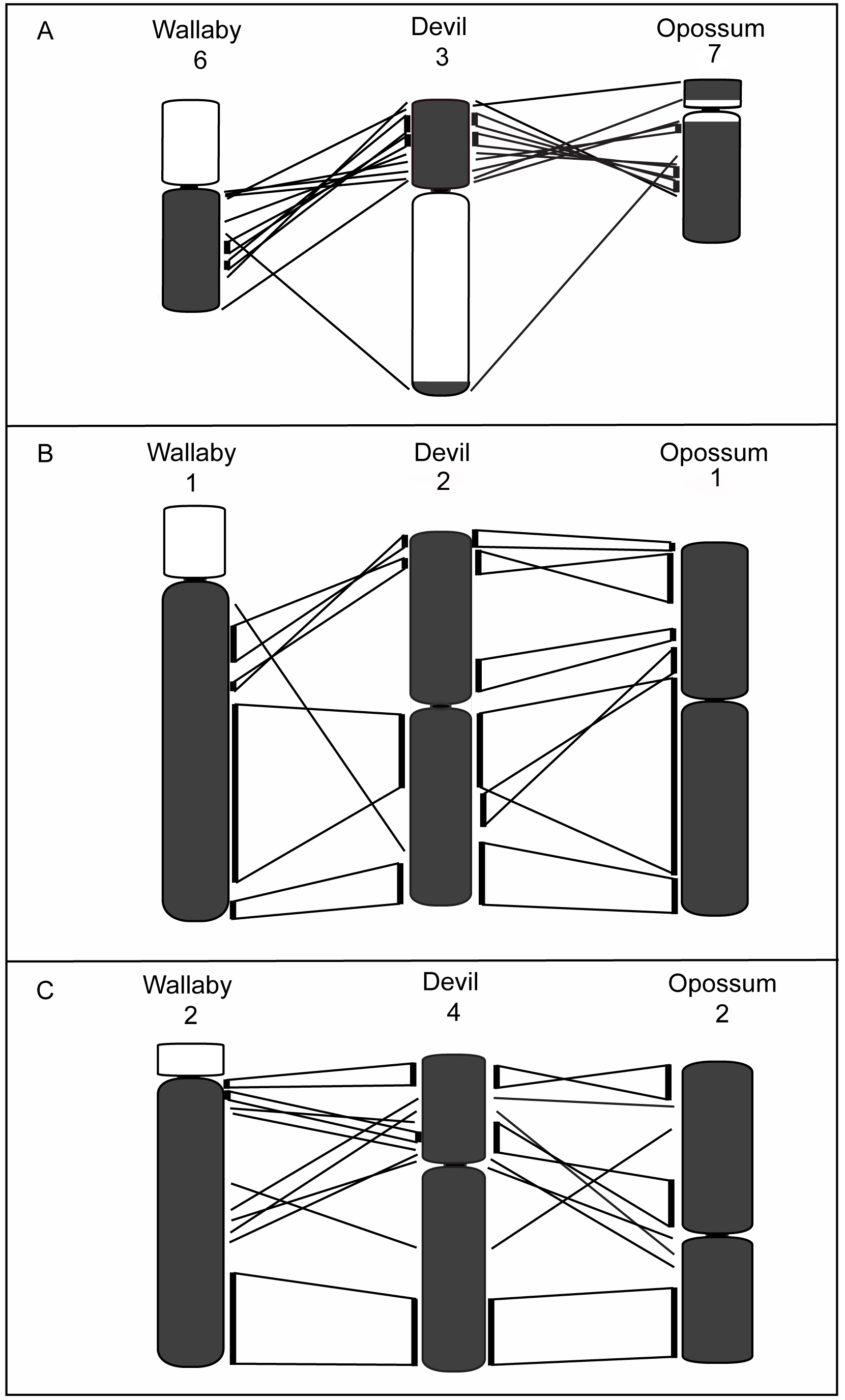

Supplement: Figure S2 — Comparison of gene arrangement between devil, wallaby and opossum chromosomes. (A) Gene order for the grey shaded region on devil chromosome 3 is considerably rearranged between species. (B) Devil chromosome 2 has large regions conserved in gene order between wallaby and opossum. (C) Devil chromosome 4 has a few blocks of genes conserved in gene order between wallaby and opossum. Opossum chromosome 2 has been inverted to make it easier to illustrate the conserved gene blocks. (TIF) [file pgen.1002483.s002.tif]

**Hoechst**

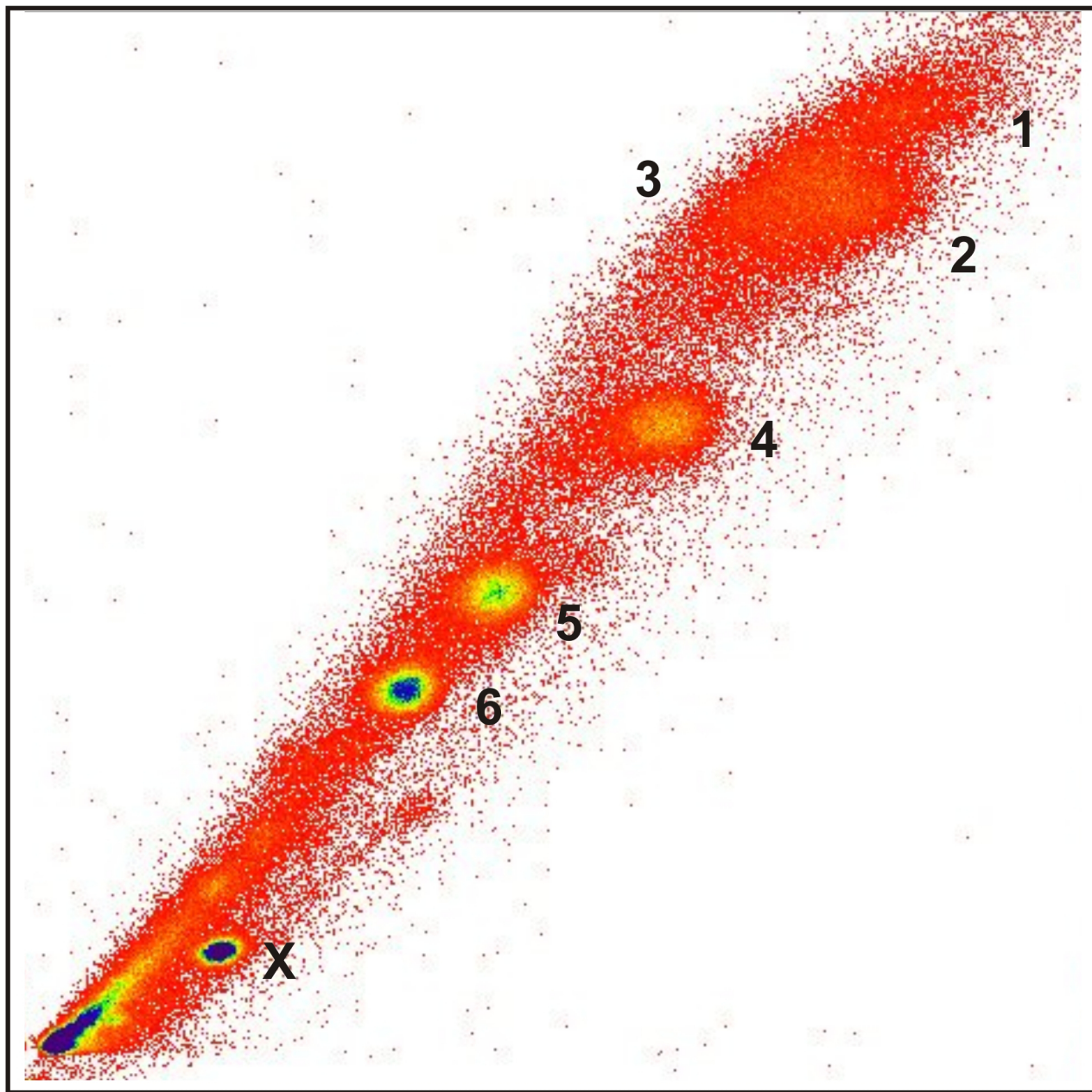

**Chromomycin**

Supplement: Figure S3 — Flow karyotype of Sarcophilus harrisii. (PDF) [file pgen.1002483.s003.pdf]

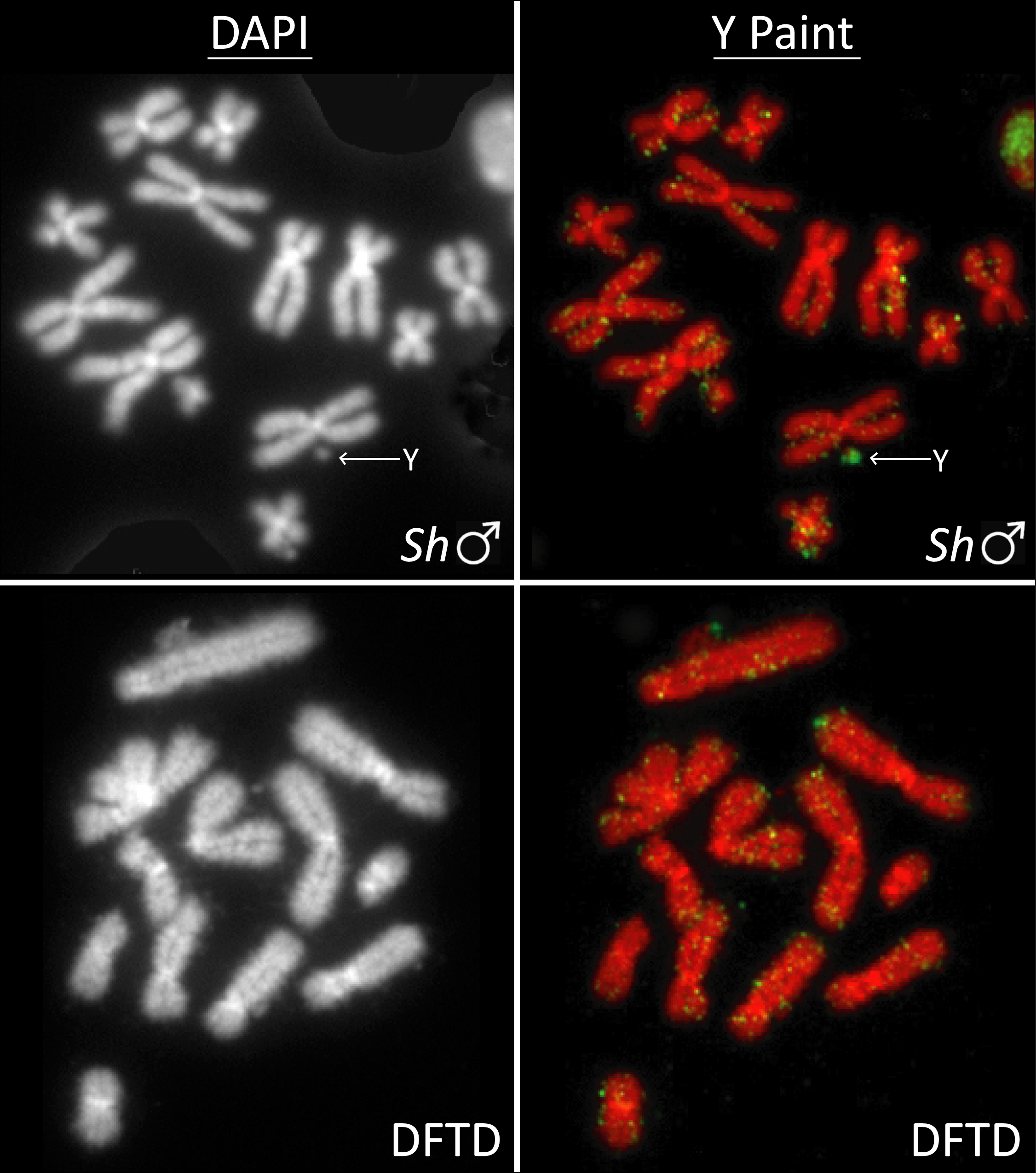

Supplement: Figure S5 — Chromosome painting using the microdissected Y chromosome on normal and DFTD chromosomes. A DAPI stained image of the chromosomes is shown on the left and hybridisation with the Y chromosome paint on the right. A clear hybridisation signal is evident on the Y chromosome on the normal male metaphase spread but not on DFTD chromosomes. (TIF) [file pgen.1002483.s005.tif]

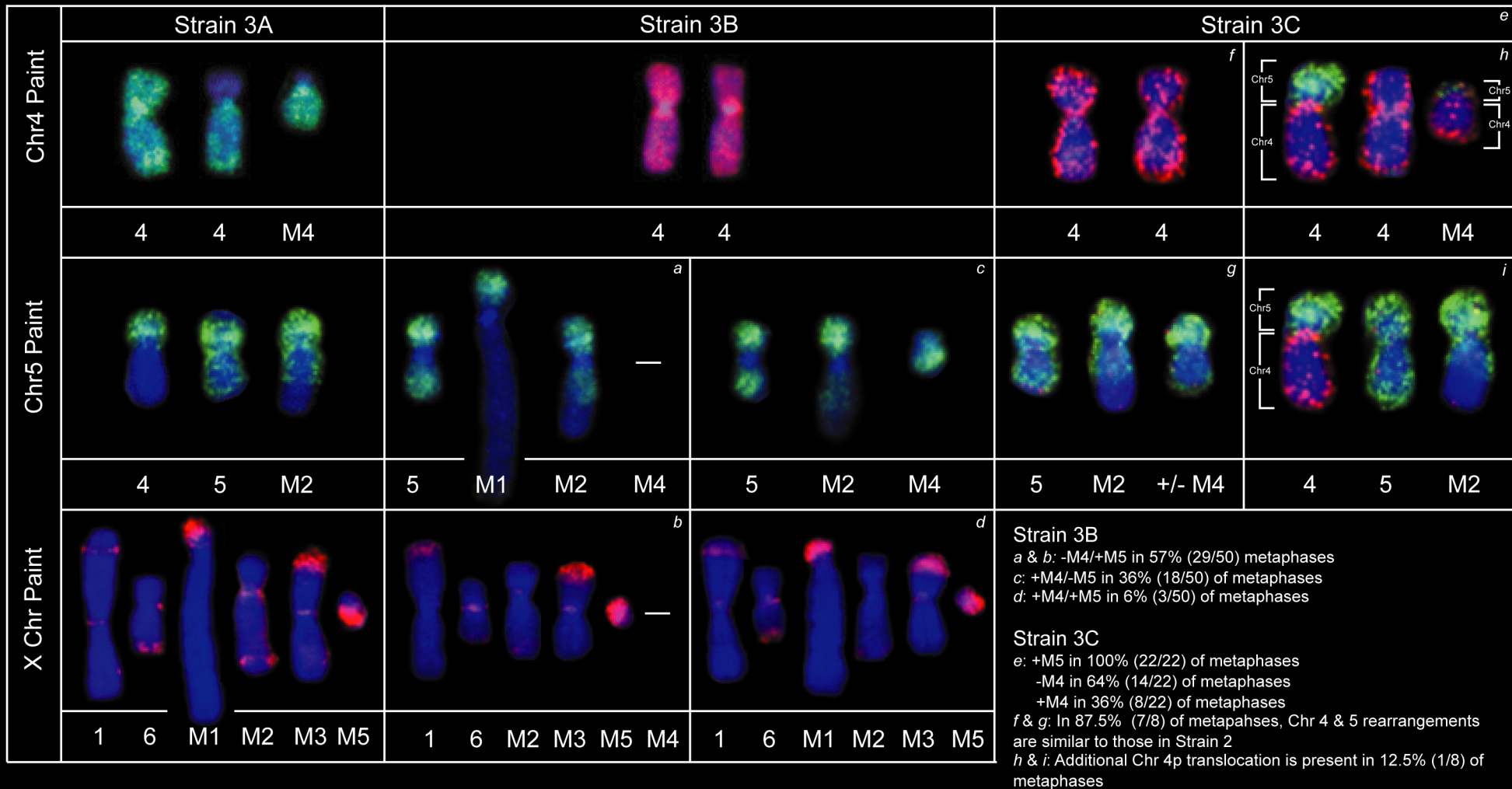

Supplement: Figure S6 — A summary of the chromosome painting differences between the three different Strain 3s. Differences between Strains 3A, 3B and 3C were detected with paints for chromosomes 4, 5 and X, and substrains of 3B and 3C were observed. (PDF) [file pgen.1002483.s006.pdf]

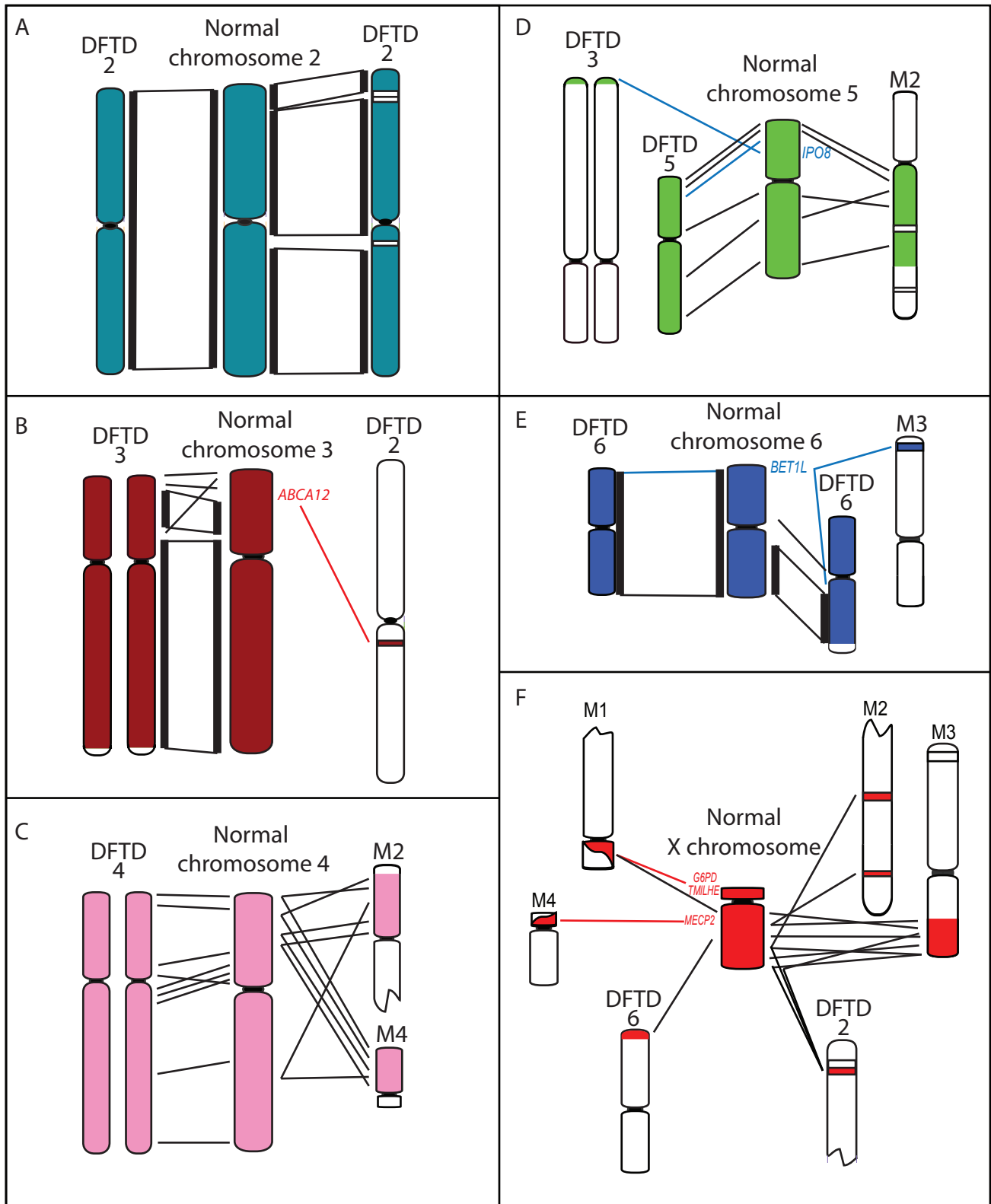

Supplement: Figure S8 — A comparison of gene arrangement on the normal devil chromosomes with arrangement observed on DFTD Strain 1 chromosomes 2 (A), 3 (B), 4 (C), 5 (D), 6 (E) and X (F). Genes in red are present in only one copy in DFTD and genes in blue are present in 3 copies. (PDF) [file pgen.1002483.s008.pdf]
