# Supplementary material for: Genomic Restructuring in the Tasmanian Devil Facial Tumour: Chromosome Painting and Gene Mapping Provide Clues to Evolution of a Transmissible Tumour
Source: PLoS Genet. 2012 Feb 16;8(2):e1002483. doi: 10.1371/journal.pgen.1002483 (PMC3280961; doi:10.1371/journal.pgen.1002483)
Supplement: Figure S4 — Information on Strains used in this study. The locations of where samples for each strain were collected are indicated on the map of Tasmania. Additional information, such as the sex and chromosome paints used on each sample, is indicated in the table below the map. (PDF) [file pgen.1002483.s004.pdf]

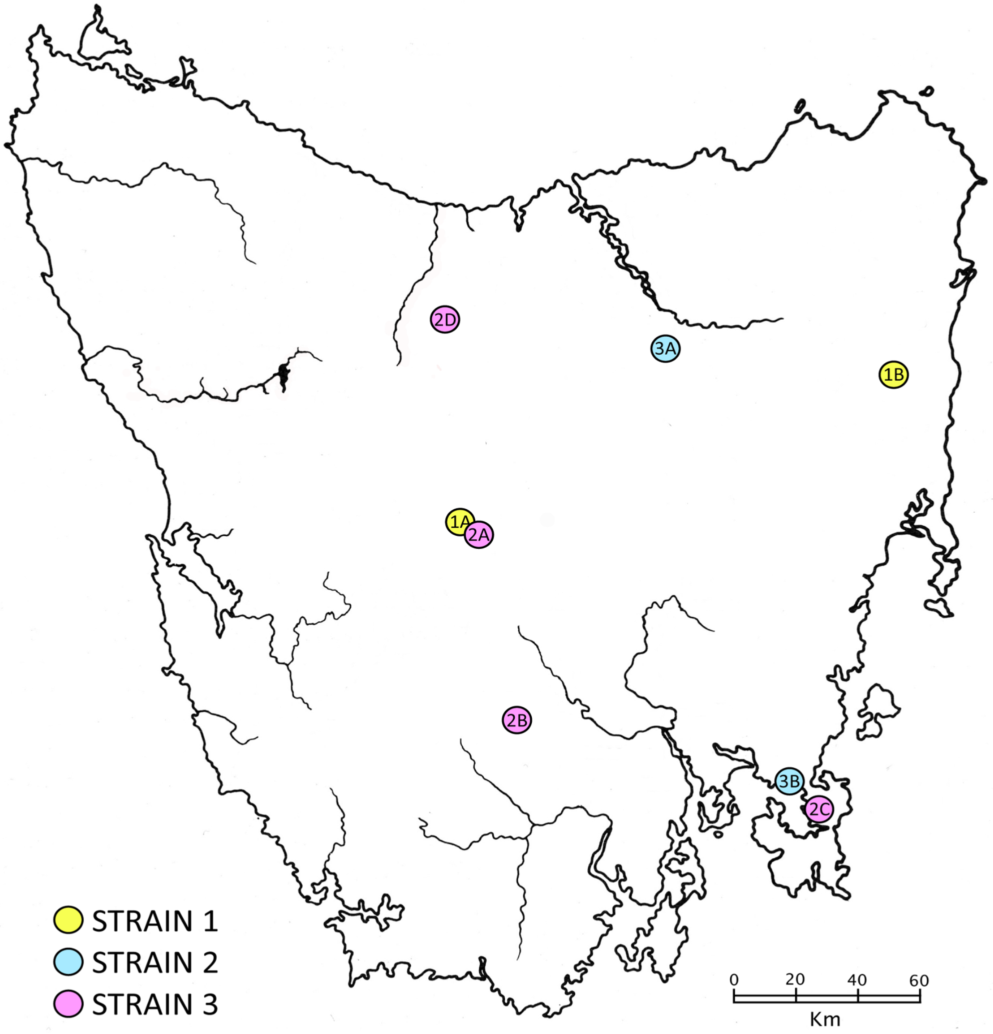

STRAIN	YEAR SAMPLED	SEX	AGE	LOCATION	ACCESSSION #	PAINTS USED	USED FOR GENE MAPPING
1A	2004	F	3	Bronte Park	04.3089	All	
1B	2006	F	1	St Marys	06.2887	1, 2, 5, 6, X	YES
2A	2006	F	2	Launceston	06.1926	All	
2B	2006	M	5	Dunally	06.0428	Chr 4	YES
3A	2005	F	1	Bronte Park	05.2748	All	YES
3B	2005	M	3	Fentonbury	05.2569	All	YES
3C	2006	M	3	Forestier	06.2109	A, B, D, E	YES
3D	2006	F	3	Sheffield	06.1645	6, X	
