# Supplementary material for: Genomic Restructuring in the Tasmanian Devil Facial Tumour: Chromosome Painting and Gene Mapping Provide Clues to Evolution of a Transmissible Tumour
Source: PLoS Genet. 2012 Feb 16;8(2):e1002483. doi: 10.1371/journal.pgen.1002483 (PMC3280961; doi:10.1371/journal.pgen.1002483)
Supplement: Figure S7 — Images of the chromosome 4 and 5 paints on metaphase spreads from a normal female and DFTD tumour strain 3. (PDF) [file pgen.1002483.s007.pdf]

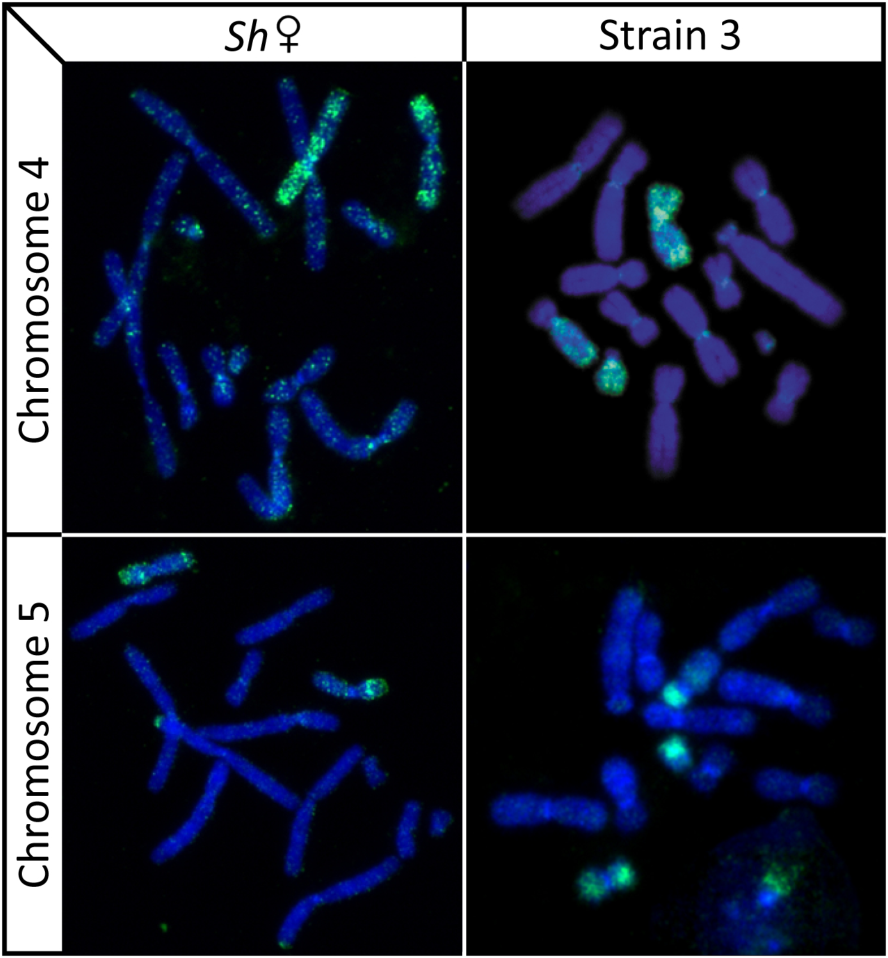

Sh♀
Strain 3
Chromosome 4
Chromosome 5
