# Supplementary material for: Genomic Restructuring in the Tasmanian Devil Facial Tumour: Chromosome Painting and Gene Mapping Provide Clues to Evolution of a Transmissible Tumour
Source: PLoS Genet. 2012 Feb 16;8(2):e1002483. doi: 10.1371/journal.pgen.1002483 (PMC3280961; doi:10.1371/journal.pgen.1002483)
Supplement: Table S2 — Success rate of overgos used for library screening. (DOCX) [file pgen.1002483.s010.docx]

**Table S2**: Success rate of overgos used for library screening.

| Chromosome | Number of overgos used for screening | Successful overgos | Success rate (%) |
| --- | --- | --- | --- |
| 1 | 28 | 26 | 93 |
| 2 | 16 | 15 | 93 |
| 3 | 28 | 23 | 82 |
| 4 | 18 | 15 | 83 |
| 5 | 25 | 6 | 24 |
| 6 | 6 | 5 | 83 |
| X | 22 | 15 | 68 |
| Total | 143 | 105 | 73 |
