# Supplementary figures and images for: Correction: Genomic Restructuring in the Tasmanian Devil Facial Tumour: Chromosome Painting and Gene Mapping Provide Clues to Evolution of a Transmissible Tumour
Source: PLoS Genet. 2014 Oct 31;10(10):e1004840. doi: 10.1371/journal.pgen.1004840 (PMC4215842; doi:10.1371/journal.pgen.1004840)

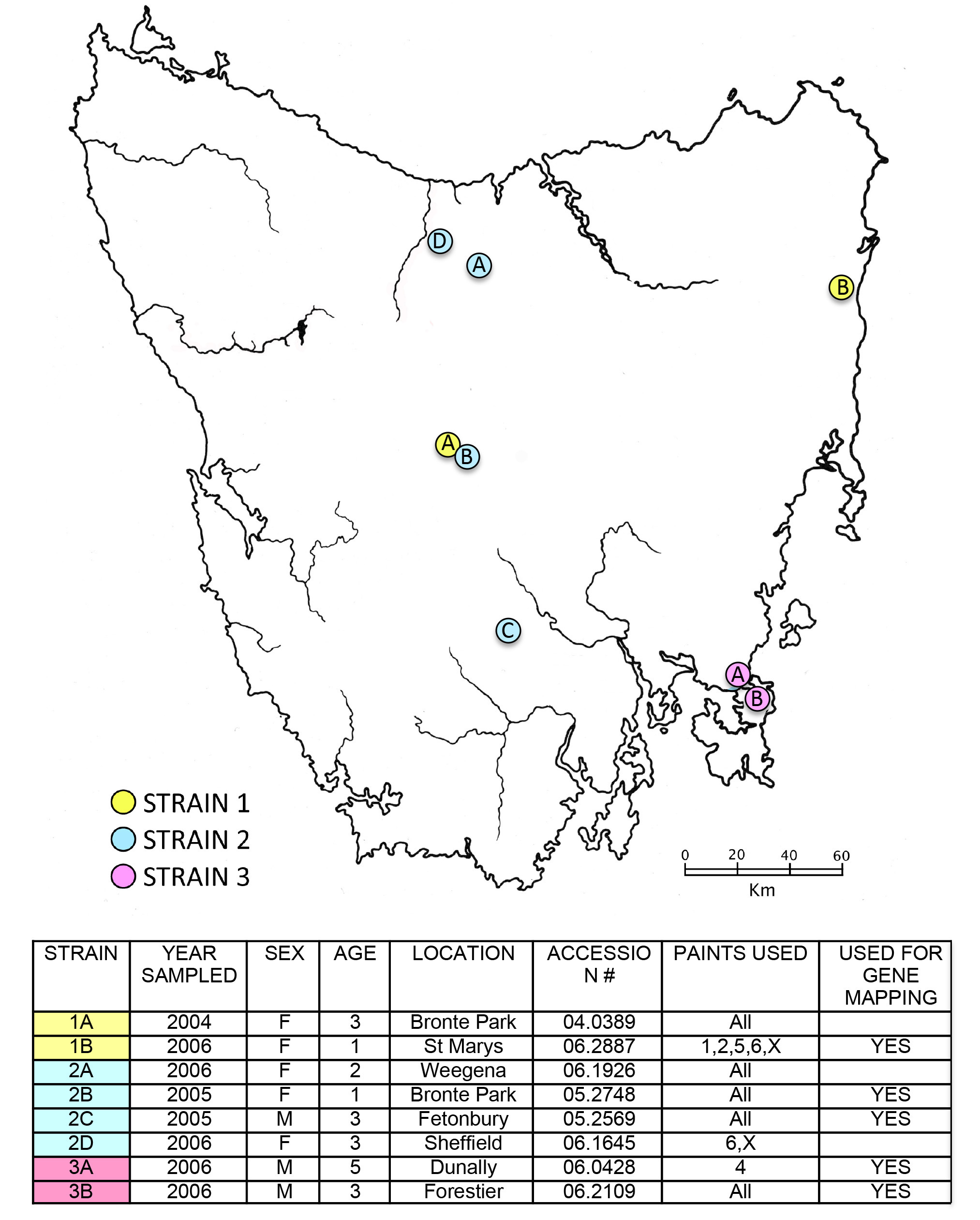

Supplement: Figure S4 — Information on Strains used in this study. The locations of where samples for each strain were collected are indicated on the map of Tasmania. Additional information, such as the sex and chromosome paints used on each sample, is indicated in the table below the map. (JPG) [file pgen.1004840.s001.jpg]

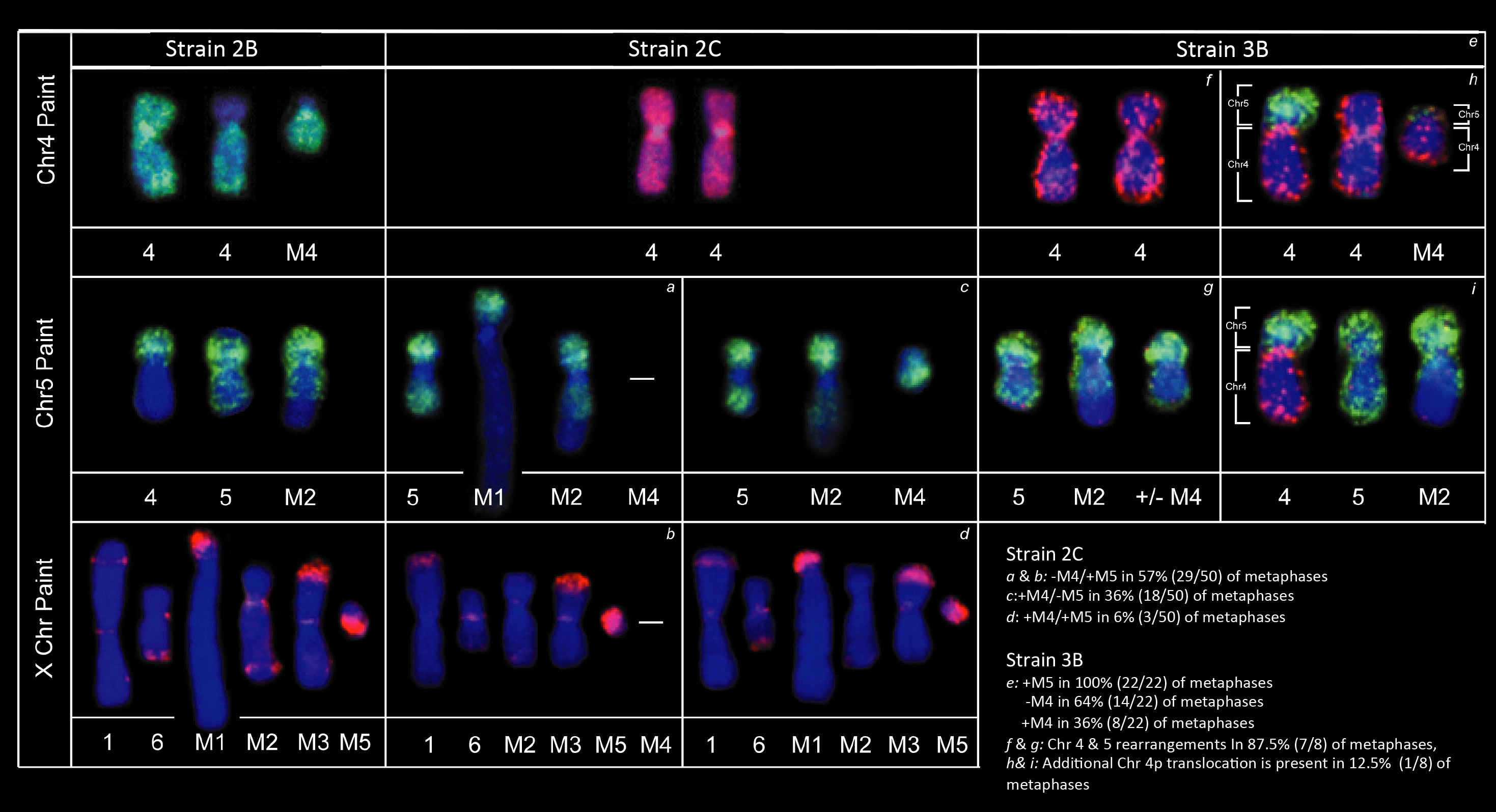

Supplement: Figure S6 — A summary of the chromosome painting differences between strains (in addition to those depicted in Figure 4). Differences between Strains 2B, 2C and 3B were detected with paints for chromosomes 4, 5 and X, and substrains of 2C and 3B were observed. (JPG) [file pgen.1004840.s002.jpg]

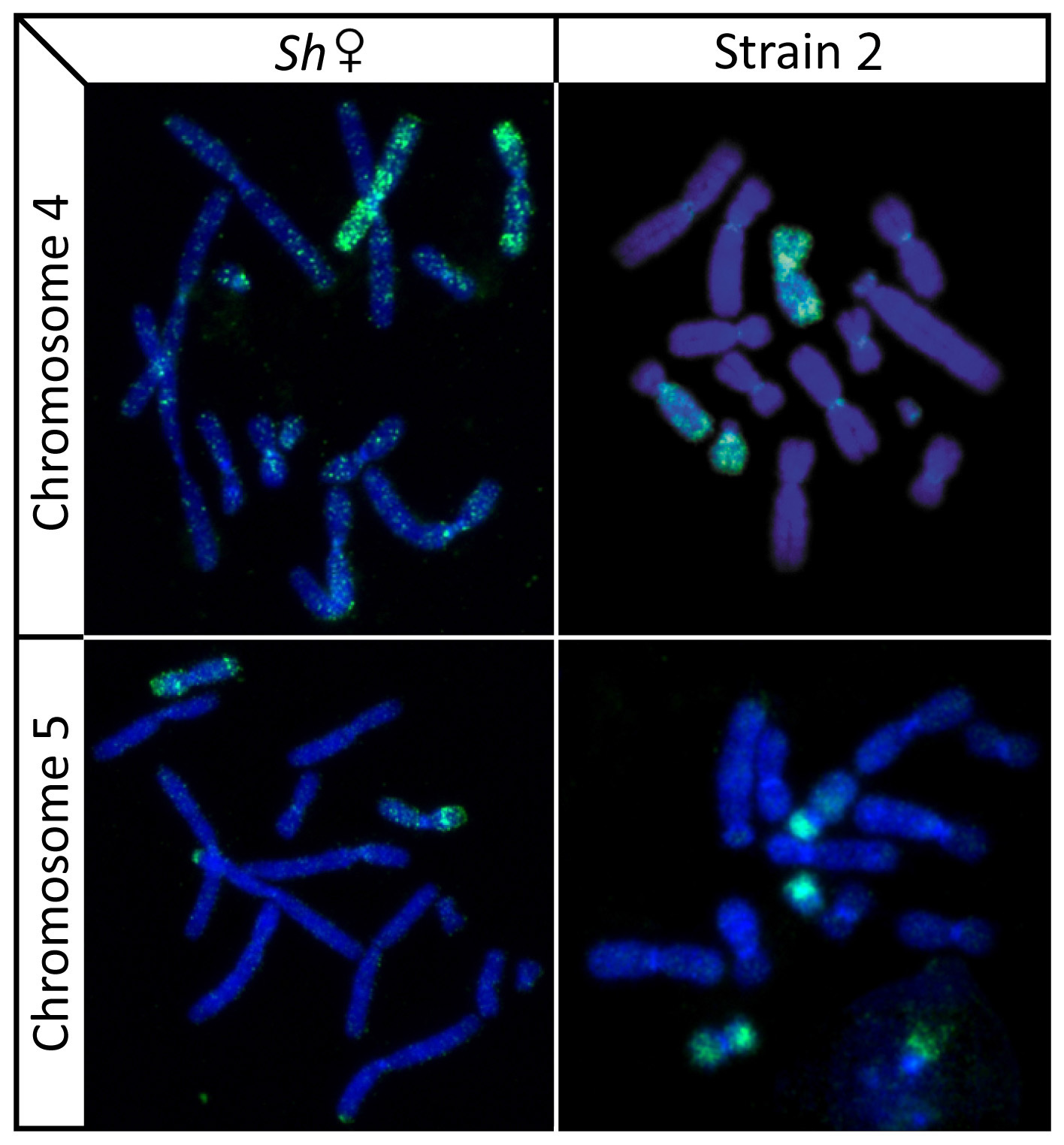

Supplement: Figure S7 — Images of the chromosome 4 and 5 paints on metaphase spreads from a normal female and DFTD tumour strain 2. (JPG) [file pgen.1004840.s003.jpg]
